# Supplementary material for: Genome-wide DNA methylation changes in human spermatogenesis
Source: Am J Hum Genet. 2024 May 16;111(6):1125–39. doi: 10.1016/j.ajhg.2024.04.017 (PMC11179423; doi:10.1016/j.ajhg.2024.04.017)
Supplement: Document S1. Figures S1–S6 [file mmc1.pdf]

The American Journal of Human Genetics, Volume *111*

Supplemental information

Genome-wide DNA methylation changes

in human spermatogenesis

Lara M. Siebert-Kuss, Verena Dietrich, Sara Di Persio, Jahnavi Bhaskaran, Martin Stehling, Jann-Frederik Cremers, Sarah Sandmann, Julian Varghese, Sabine Kliesch, Stefan Schlatt, Juan M. Vaquerizas, Nina Neuhaus, and Sandra Laurentino

Supplemental information

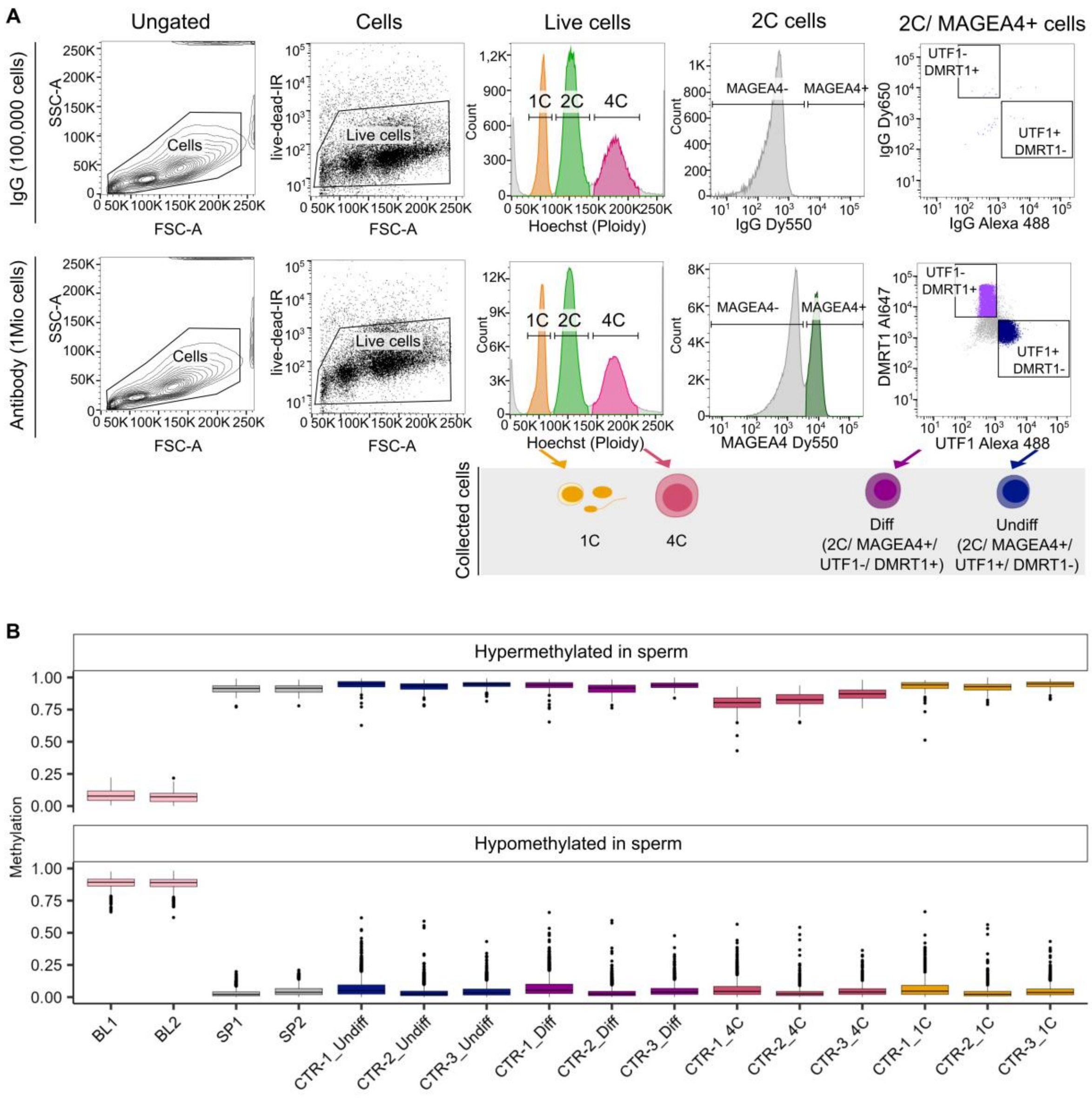

Figure S1: Sorting strategy and quality check of sorted cells. A Sorting strategy for the different germ cell types represented by 100,000 cells in the IgG and 1 million (Mio) cells in the antibodystained cells. IgG control showed no positive signal for MAGEA4 within the 2C cells and no signal for UTF1 and DMRT1 within the 2C/MAGEA4+ cells. **B** Box plots display methylation of sperm and blood[60] and each control (CTR) sample in 2,761 sperm-soma DMRs of which 121 are hypermethylated and 2,640 are hypomethylated in sperm. Data are represented as median (center line), upper/ lower quartiles (box

limits), 1.5 x interquartile range (whiskers). Undiff = undifferentiated spermatogonia, Diff = differentiating spermatogonia, 4C = primary spermatocytes, 1C = spermatids/sperm.

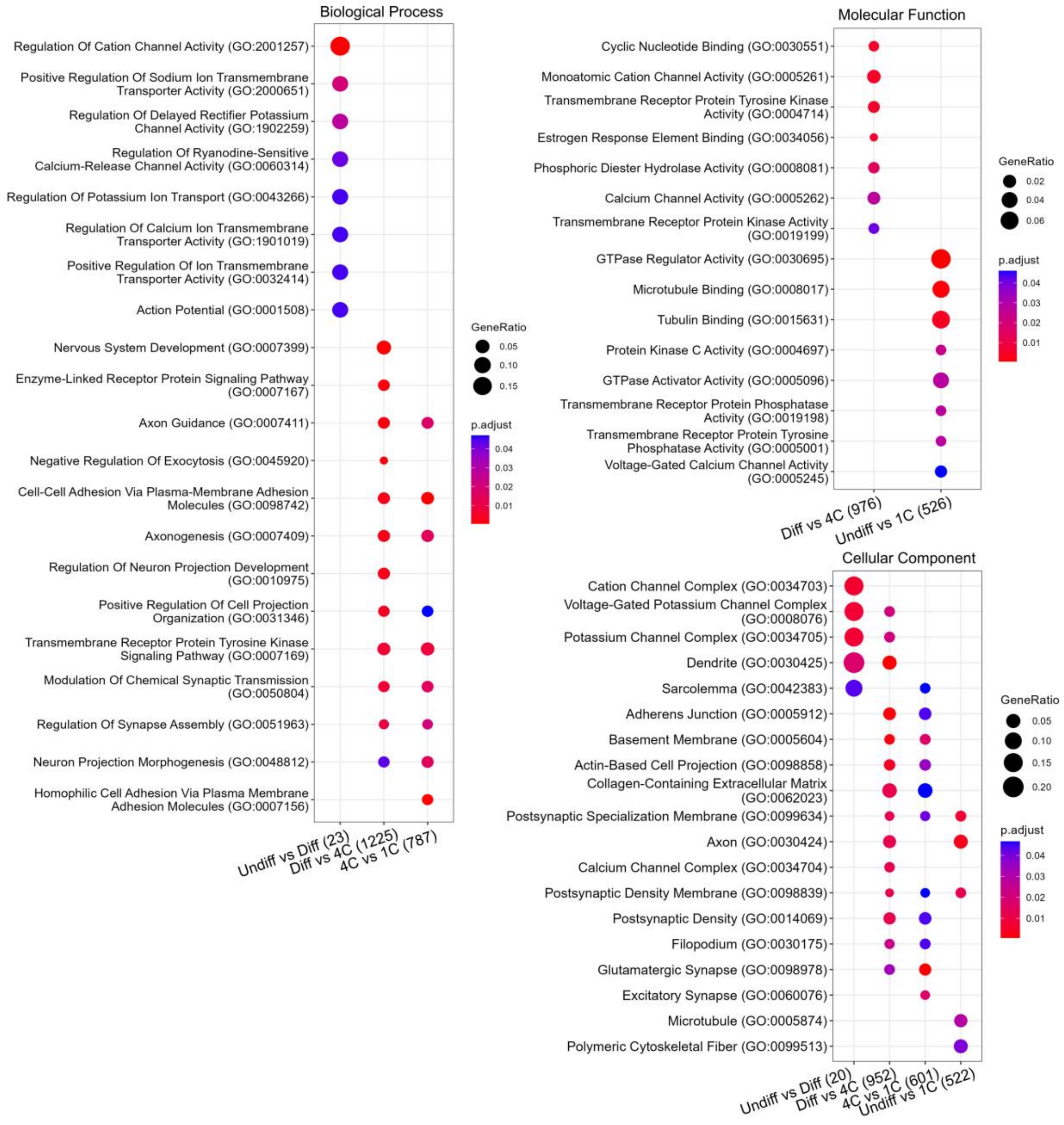

Figure S2: GO term enrichment analysis of biological processes, molecular function and cellular components of the control (CTR) differentially methylated regions (DMRs). All DMR group comparisons were assessed and significant results of the top 8 terms with the lowest p-values are displayed. P-values were adjusted for multiple testing with Benjamini-Hochberg correction. Undiff =

undifferentiated spermatogonia, Diff = differentiating spermatogonia, 4C = primary spermatocytes, 1C = spermatids/sperm.

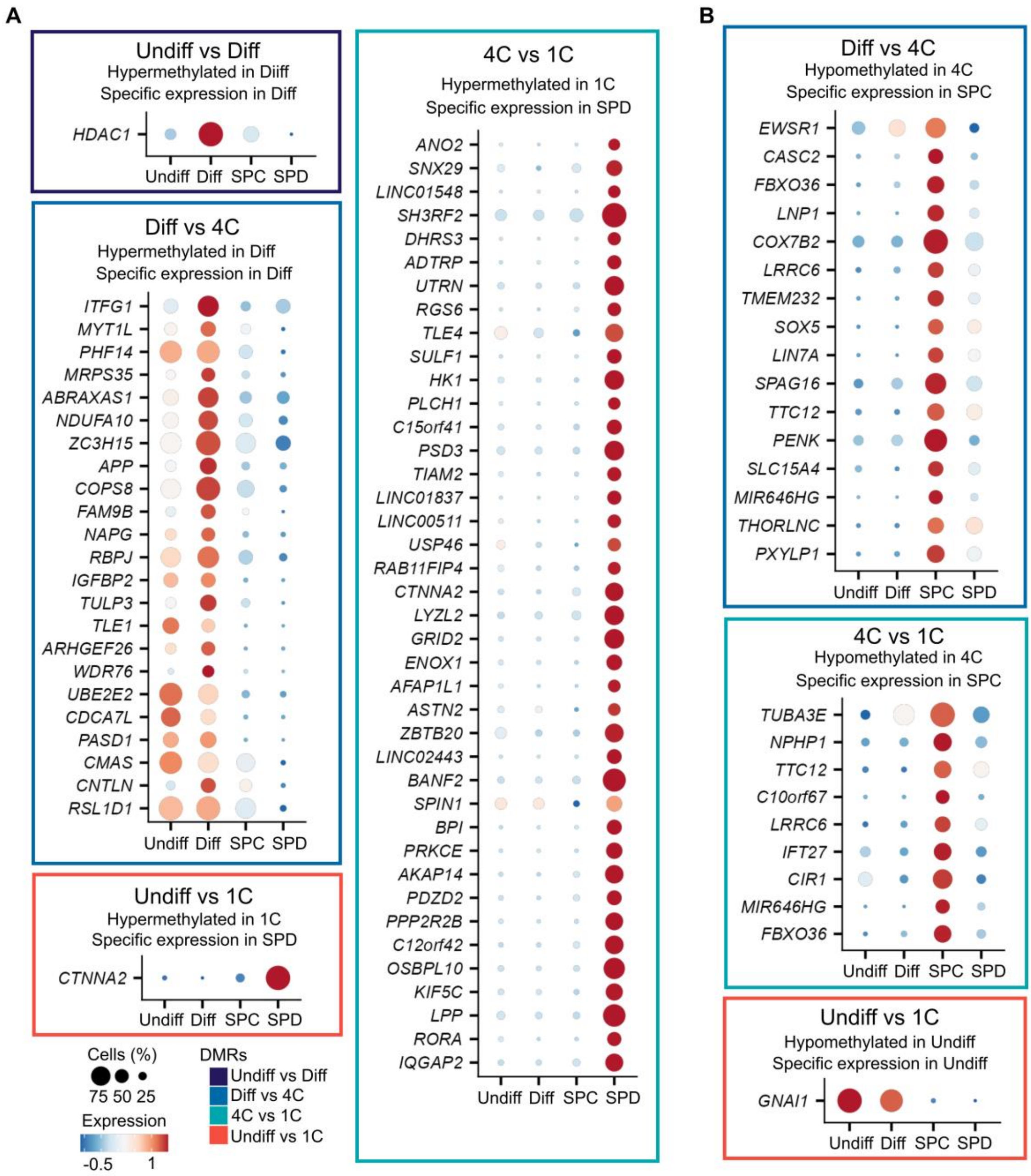

Figure S3: Differentially methylated region (DMR)-associated genes with germ cell type-specific expression. A Hypermethylated and **B** hypomethylated DMR-associated genes of the different group comparisons with specific expression in the respective germ cell types[16] are depicted in dot plots. Undiff = undifferentiated spermatogonia, Diff = differentiating spermatogonia, 4C = primary spermatocytes, 1C = spermatids/sperm, SPC = spermatocytes, SPD = spermatids.

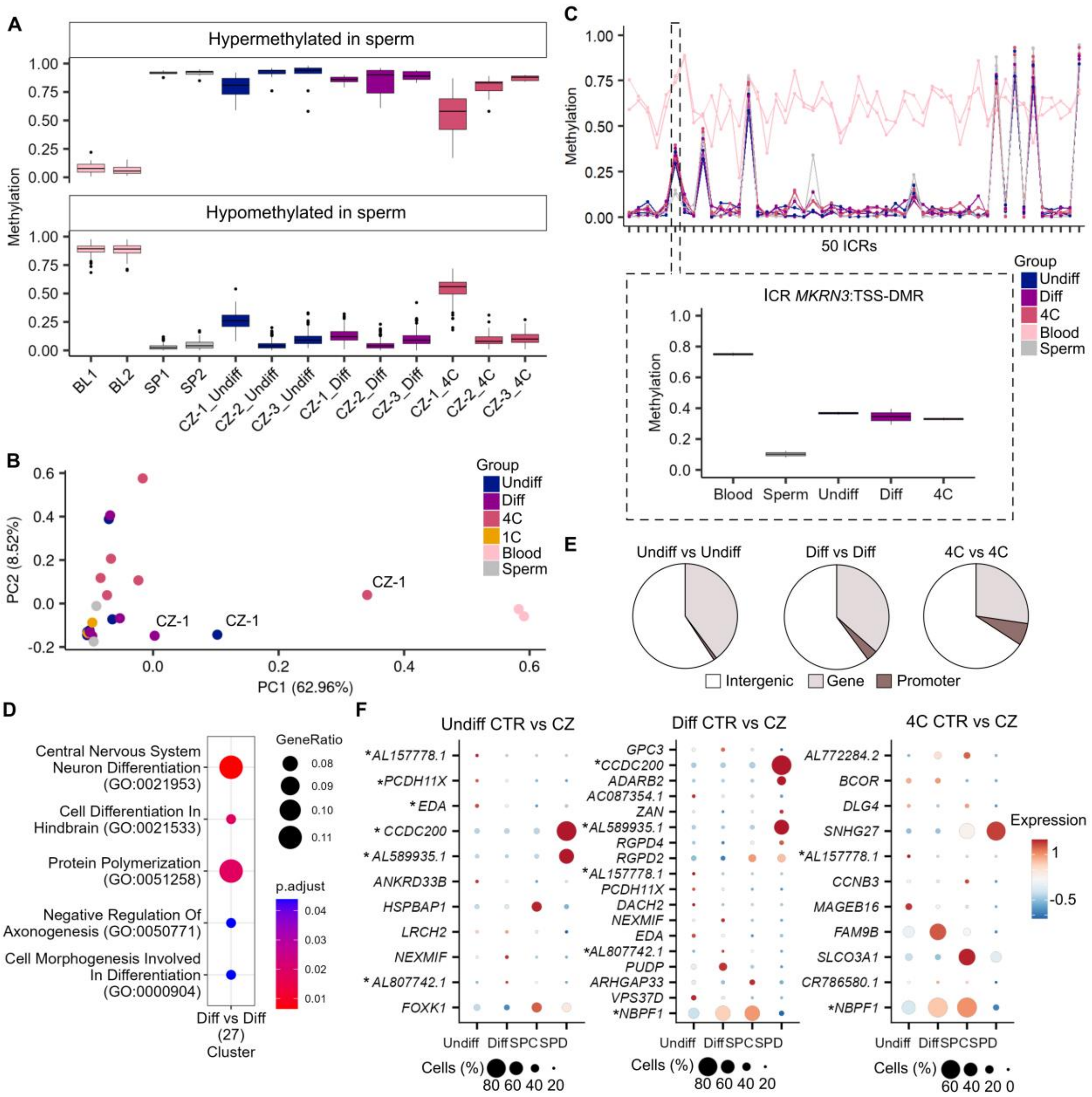

Figure S4: Purity check of the cryptozoospermic samples and features of control (CTR)/cryptozoospermic (CZ) differentially methylated region (DMR)-associated genes. A Box plots display methylation of sperm and blood[60] and each CZ sample in 2,761 sperm-soma DMRs. **B** Principal component analysis (PCA) of 2,521 CpGs of the imprinting control regions (ICRs) depicts clustering of all CTR samples and CZ-2 and CZ-3 samples together with sperm, whereas CZ-1 clustered towards blood, explaining 62.96% of variance (PC1). **C** Lineplots show the mean methylation in the 50 ICRs (Table S8) for CZ-2 and CZ-3 in undifferentiated spermatogonia, differentiating spermatogonia, and primary spermatocytes compared to blood and sperm samples[60]. Box plots display the methylation of

MKRN3:TSS-DMR in the respective cell types. Data are represented as median (center line), upper/ lower quartiles (box limits), 1.5 x interquartile range (whiskers). **D** Piecharts show the annotation of the CTR/CZ DMRs for genes, promoters and intergenic regions. **E** GO term enrichment analysis for biological processes in the CTR/CZ DMRs. The top 5 results are shown. **F** Dot plots showing DMR-associated genes with germ cell-type specific expression[16]. Genes marked with asterisks are present in more than one group. Undiff = undifferentiated spermatogonia, Diff = differentiating spermatogonia, 4C = primary spermatocytes, SPC = spermatocytes, SPD = spermatids. Panel A assembled with BioRender.com.

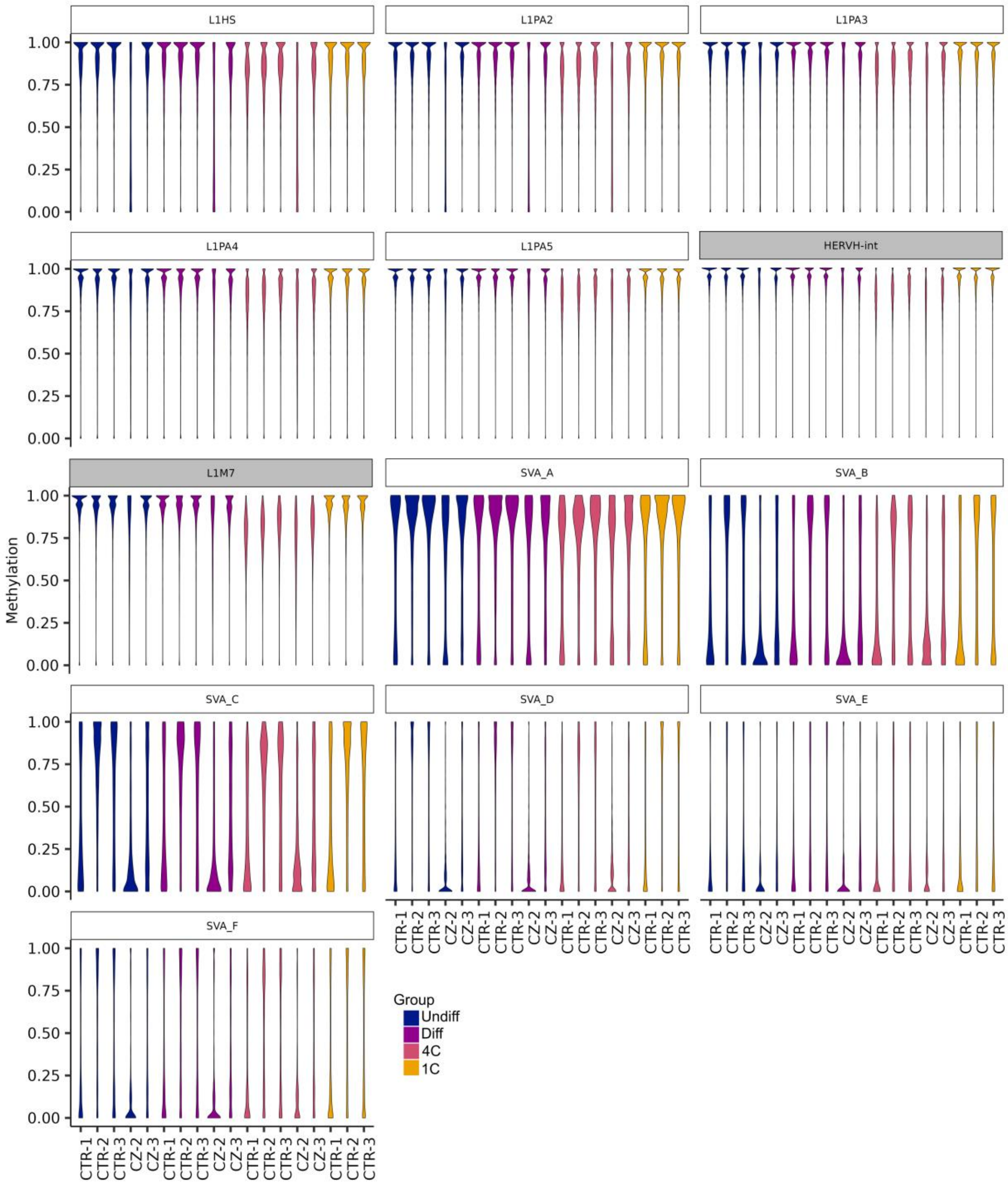

Figure S5: DNA methylation levels of transposable elements (TEs) per control (CTR) and cryptozoospermic (CZ) samples. Violin plots showing the CpG methylation of evolutionarily younger (white boxes: L1Hs, L1PA2-5, and SVA A/B/C/D/E/F) and older (grey boxes: HERVH-int and L1M7) TEs

per CTR and CZ samples. Undiff = undifferentiated spermatogonia, Diff = differentiating spermatogonia, 4C = primary spermatocytes, 1C = spermatids/sperm.

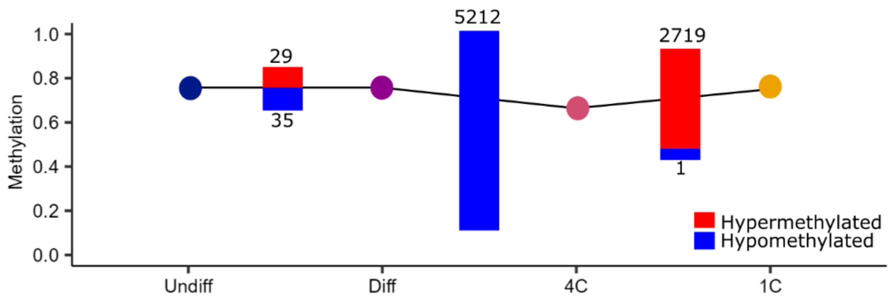

Figure S6: Schematic summary of DNA methylation changes throughout normal spermatogenesis identified in this study. Mean global methylation levels are indicated by dots. Number of hyper- or hypomethylated differentially methylated regions (DMRs), compared to the more advanced germ cell type, are depicted as bars and color-coded red and blue, respectively. Undiff = undifferentiated spermatogonia, Diff = differentiating spermatogonia, 4C = primary spermatocytes, 1C = spermatids/sperm.
