# Supplementary material for: Genome-wide DNA methylation changes in human spermatogenesis
Source: Am J Hum Genet. 2024 May 16;111(6):1125–39. doi: 10.1016/j.ajhg.2024.04.017 (PMC11179423; doi:10.1016/j.ajhg.2024.04.017)
Supplement: Document S2. Article plus supplemental information [file mmc12.pdf]

ARTICLE

Genome-wide DNA methylation changes in human spermatogenesis

Graphical abstract

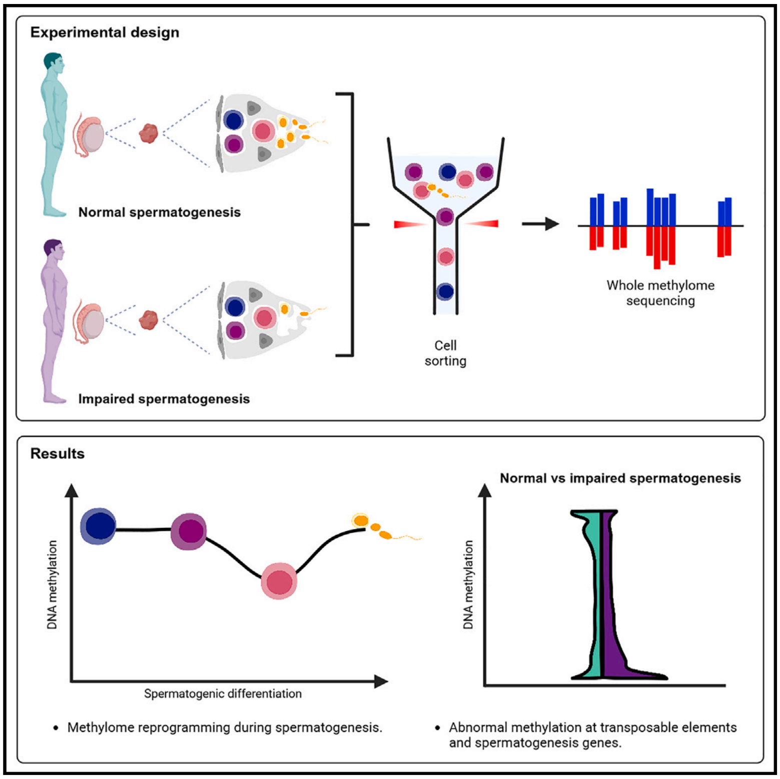

Authors

Lara M. Siebert-Kuss, Verena Dietrich, Sara Di Persio, ..., Juan M. Vaquerizas, Nina Neuhaus, Sandra Laurentino

Correspondence

sandra.laurentino@ukmuenster.de

Correct DNA methylation patterns are essential for sperm function. We evaluated genome-wide DNA methylation changes throughout human adult germ cell differentiation. In normal spermatogenesis, we found remodeling of the methylome during spermatogenesis. Impaired spermatogenesis was associated with abnormal methylation, especially affecting some types of transposable elements.

Siebert-Kuss et al., 2024, The American Journal of Human Genetics *111*, 1125–1139
June 6, 2024 © 2024 The Author(s).
https://doi.org/10.1016/j.ajhg.2024.04.017

CellPress

Genome-wide DNA methylation changes in human spermatogenesis

Lara M. Siebert-Kuss,[1,7] Verena Dietrich,[2,7] Sara Di Persio,[1] Jahnavi Bhaskaran,[3,4,5] Martin Stehling,[5] Jann-Frederik Cremers,[6] Sarah Sandmann,[2] Julian Varghese,[2] Sabine Kliesch,[6] Stefan Schlatt,[1] Juan M. Vaquerizas,[3,4,5] Nina Neuhaus,[1,8] and Sandra Laurentino[1,8,*]

Summary

Sperm production and function require the correct establishment of DNA methylation patterns in the germline. Here, we examined the genome-wide DNA methylation changes during human spermatogenesis and its alterations in disturbed spermatogenesis. We found that spermatogenesis is associated with remodeling of the methylome, comprising a global decline in DNA methylation in primary spermatocytes followed by selective remethylation, resulting in a spermatids/sperm-specific methylome. Hypomethylated regions in spermatids/sperm were enriched in specific transcription factor binding sites for DMRT and SOX family members and spermatid-specific genes. Intriguingly, while SINEs displayed differential methylation throughout spermatogenesis, LINEs appeared to be protected from changes in DNA methylation. In disturbed spermatogenesis, germ cells exhibited considerable DNA methylation changes, which were significantly enriched at transposable elements and genes involved in spermatogenesis. We detected hypomethylation in SVA and L1HS in disturbed spermatogenesis, suggesting an association between the abnormal programming of these regions and failure of germ cells progressing beyond meiosis.

Introduction

There is growing evidence that the establishment of the male germ cell methylome is not restricted to embryonic development but continues in adulthood during spermatogenesis.[1–5] Especially in the early phases of meiosis, when replication and recombination occur, the genome is hypomethylated. This event is highly conserved between mouse and human and has been hypothesized to result from a delay in DNA methylation maintenance.[5,6] Still, there is limited information on DNA methylation during human spermatogenesis or whether disturbances in this process impact spermatogenesis and sperm function.

Epigenetic remodeling, which includes reprogramming of the methylome, is essential for cell-fate decisions in mammals.[7–9] Recently, it has been demonstrated that the impaired function of enzymes involved in the DNA methylation machinery results in a range of disturbances to spermatogenesis, including complete sterility.[10–14] In most cases of severe male infertility, sperm output is drastically reduced, and the etiology is unknown.[15] In particular, cryptozoospermia (CZ) is characterized by a drastic decline in germ cells going through meiosis and an accumulation of the most undifferentiated spermatogonia.[16] Previously, we reported aberrant DNA methylation in bulk germ cells of men displaying CZ,[3] which led us to hypothesize that aberrant DNA methylation could be the underlying cause. However, the extent to which the different germ cell types carry altered DNA methylation, the genomic regions affected, and their involvement in post-meiotic germ cell decline remain to be assessed.

Especially during meiosis, the genome heavily depends on the suppression of transposable elements (TEs). This is usually achieved by epigenetic mechanisms such as gene-silencing histone modifications, piwi-interacting RNA (piRNA) machinery, and DNA methylation.[11,17] Thus, the suppression of TEs, such as long- and short-interspersed nuclear elements (LINE/SINE), is crucial to maintaining genome integrity in the germline,[17–20] which, when lost, leads to sterility in mice.[12,21] Differential methylation at TEs or dysfunctional TE silencing pathways have been linked to male infertility.[22–26] Importantly, different TEs have different modes of DNA methylation acquisition,[27] and evolutionary younger (SINE-VNTR-Alus [SVAs]) TEs are protected from genome-wide methylome erasure during development,[28–31] indicating that these elements are especially hazardous to the genome and must be protected via DNA methylation. The protective role of DNA methylation at different TEs in germ cells during spermatogenesis and its association with male infertility, in particular during the hypomethylated phase of meiosis, remains to be elucidated.

By performing whole-methylome analysis on pure human male germ cell fractions, we uncovered that human

[1]Centre of Reproductive Medicine and Andrology, Institute of Reproductive and Regenerative Biology, University of Münster, Münster, Germany; [2]Institute of Medical Informatics, University of Münster, Münster, Germany; [3]MRC Laboratory of Medical Sciences, London, UK; [4]Institute of Clinical Sciences, Imperial College London, London, UK; [5]Max Planck Institute for Molecular Biomedicine, Münster, Germany; [6]Department of Clinical and Surgical Andrology, Centre of Reproductive Medicine and Andrology, University Hospital of Münster, Münster, Germany
[7]These authors contributed equally
[8]Senior author
*Correspondence: sandra.laurentino@ukmuenster.de
https://doi.org/10.1016/j.ajhg.2024.04.017.
© 2024 The Author(s). This is an open access article under the CC BY license (http://creativecommons.org/licenses/by/4.0/).

spermatogenesis is associated with epigenetic remodeling of the methylome. We found that the global decline in DNA methylation in primary spermatocytes is followed by selective remethylation in specific regions in spermatids/sperm, suggesting that the hypomethylated primary spermatocyte genome is not exclusively a transient side effect of DNA replication but is required for the establishment of a spermatids/sperm-specific methylome. We found significant differences in DNA methylation in germ cells of infertile men, particularly occurring in intergenic regions and TEs, and demonstrate that different TEs are differentially reprogrammed during spermatogenesis. Our study increases current evidence on the role of DNA methylation changes during human spermatogenesis and points to an association between altered DNA methylation and spermatogenic failure.

Material and methods

Ethical approval

Individuals included in this study underwent surgery for (microdissection) testicular sperm extraction (mTESE; $n = 6$) at the Department of Clinical and Surgical Andrology of the Centre of Reproductive Medicine and Andrology, University Hospital of Münster, Germany. Each individual gave written informed consent (ethical approval was obtained from the Ethics Committee of the Medical Faculty of Münster and the State Medical Board no. 2008-090-f-S), and one additional testicular sample for the purpose of this study was obtained. Tissue portions were either enzymatically digested or fixed in Bouin's solution.

Selection and clinical evaluation of the cohort

All individuals included in this study underwent full physical examination, hormonal analysis of luteinizing hormone, follicle-stimulating hormone (FSH), and testosterone (T), as well as semen analysis[32] and genetic analyses of the karyotype and screening for azoospermia factor (AZF) deletions. All subjects had normal karyotypes (46,XY) and no AZF deletions. Exclusion criteria were biopsies of testis with germ cell neoplasia, a history of cryptorchidism of the biopsied testis, and acute infections. To study qualitatively and quantitatively normal spermatogenesis, we collected biopsies from control subjects (CTR) that were diagnosed with obstructive azoospermia due to anejaculation (CTR-1) or a congenital bilateral absence of the *vas deferens* due to *CFTR* gene mutations (CTR-2 and CTR-3). No sperm was found in the ejaculate but sperm was found after mechanical dissection of the testicular tissues. To study severely disturbed spermatogenesis, we collected biopsies from cryptozoospermic subjects (CZ-1, CZ-2, and CZ-3) that were diagnosed with hypergonadotropic oligoasthenoteratozoospermia and presented <0.1 million sperm in the ejaculate after centrifugation. All subjects with CZ had elevated FSH levels (Table S1).

Histological analysis of the human testicular biopsies

As a routine diagnostic procedure in our clinic, two testicular biopsies per testis were fixed in Bouin's solution, the tissues were washed in 70% ethanol, embedded in paraffin, and sectioned at 5 μm. For histological evaluation, two independent testicular sections from each testis were stained with periodic acid-Schiff/hematoxylin and were evaluated based on the Bergmann and Kliesch scoring method[33] as previously described.[34]

Preparation of single-cell suspensions from testicular biopsies

For the extraction of pure germ cell subtypes, testicular biopsies were digested into a single-cell suspension as previously published.[16] The digestion was based on mechanically chopping up the testicular tissue with a sterile blade into ~1 mm^3 pieces and a two-step enzymatic incubation: first, with MEMα (ThermoFisher scientific, Gibco, Cat# 22561021) with 1 mg/mL collagenase IA (Merck/Sigma Aldrich, Cat# C9891) at 37°C for 10 min and, second, with Hank's balanced salt solution (HBSS) containing 4 mg/mL trypsin (Thermo Fisher Scientific, Gibco, Cat# 27250018) and 2.2 mg/mL of DNase I (Merck/Sigma-Aldrich, Cat# DN25) at 37°C for 8–10 min and strong pipetting in between. Each enzymatic reaction was stopped by adding MEMα supplemented with 10% fetal bovine serum (FBS) (Merck, Cat# S0615) and 1% penicillin/streptomycin (Thermo Fisher Scientific, Gibco, Cat# 15140-148), and the supernatant was discarded after centrifugation. Finally, cells were washed three times, and the cell suspension was then depleted from erythrocytes by incubation in hemolysis buffer (0.83% NH4Cl solution) for 3 min. The reaction was stopped as described above. Cell debris was removed by filtering the cell suspension through a 70 μm sterile CellTrics filter (Sysmex). We used the trypan blue exclusion method for quantification of the obtained cell numbers. We incubated the cells (1 million cells/1 mL HBSS) obtained after digestion with 1 μL Near-IR fluorescent reactive dye LIVE/DEAD Fixable Dead Cell Stain Kit (Invitrogen; Cat# L34961; Ex: 633/635 nm; Em: 775 nm) for 30 min on ice and stopped the reaction by addition of 1 mL HBSS supplemented with 5% FBS. After centrifugation, unspecific antibody binding sites were blocked by incubation of the cells with HBSS containing 5% FBS for 45 min on ice. Following centrifugation, cells were fixed in Fix and Perm solution A for 30 min at room temperature, and the reaction was stopped as outlined above. After centrifugation, cell membranes were permeabilized by incubation with Fix and Perm solution B for 30 min at room temperature. After centrifugation 20% of cells were incubated with fluorophores-conjugated, unspecific immunoglobulin G (IgG) as negative controls, namely mIgG-AI647 (1:200, BioLegend, Cat# 400130), mIgG-Dy550 (1:20, Biolegend Cat# 400166), and mIgG-Dy488 (1:20, BioLegend Cat# 400166). The remaining cells were incubated with fluorophores-conjugated primary antibodies at room temperature for 1 h against DMRT1-AI647 (1:200, Santa Cruz, Cat# sc-377167 AF647), MAGEA4-Dy550 (1:20, Prof. G. C. Spagnoli, University Hospital of Basel, CH, conjugated), and UTF1-Dy488 (1:20, Merck/Millipore, Cat# MAB4337, conjugated). We used the Dylight 488 and 550 labeling kits (Thermo Fisher, Cat# 53025 and CAT#84531) for conjugation of the IgGs, MAGEA4, and UTF1 antibodies, respectively. The antibody binding reaction was stopped by adding HBSS supplemented with 5% FBS. After centrifugation, cells were resuspended in HBSS containing 5% FBS, and Hoechst (NucBlue Live ReadyProbes Reagent Protocol, R37605, Thermo Fisher) was added to distinguish the DNA contents of the cells.

Fluorescence-activated cell sorting analyses for isolation of human male germ cells

For extraction of the different germ cell fractions, we applied a multi-parameter fluorescence-activated cell sorting (FACS) strategy

on the BD FACS Aria Fusion with FACSDiva software (v 8.02). We gated for cells and live cells based on the Near-IR fluorescent reactive dye LIVE/DEAD (LIVE/DEAD Fixable Near-IR Dead Cell Stain Kit, Invitrogen, L10119). Spermatogonia were identified and gated within the diploid (2C) cells that were positive for the pan-spermatogonial marker MAGEA4. The 2C/MAGEA4+ cells were further divided into undifferentiated spermatogonia (Undiff) and differentiating spermatogonia (Diff) by gating for UTF1^{+}/DMRT1^{-} and UTF1^{-}/DMRT1^{+} cells, respectively. Primary spermatocytes and spermatids/sperm were isolated and gated based on their DNA content for 4C and 1C cells, respectively. Cells were sorted with a 85 μm nozzle and collected in 200 μL HBSS containing 5% FBS. FACS data were analyzed using the FlowJo software (v 10.8.1).

DNA isolation and enzymatic conversion of sorted testicular germ cells

DNA was isolated from fixed and sorted cells using the MasterPure DNA purification kit (MC85200, Lucigen, LGC Ltd, Teddington, UK) following the manufacturer's protocol. We incubated the cells for 20 min at 90°C prior to incubation with proteinase K at 65°C for 1 h. DNA concentration was measured using Qubit dsDNA HS Assay Kit (life technologies) and a fluorescence plate reader (FLUOstar Omega, BMG Labtech, Germany). Enzymatic conversion was performed using the NEBNext Enzymatic Methyl-seq Conversion Module (New England BioLabs, Cat# E7125S) according to the manufacturer's protocol.

Whole-genome enzymatic methylation sequencing (EM-seq)

EM-seq libraries were prepared individually from the sorted testicular germ cell fractions of each CTR and CZ sample ($n = 21$) using 10–200 ng of DNA supplemented with 0.01% Lambda-/0.001% PUC-DNA. DNA was fragmented using the Bioruptor with 3 cycles of 30 s on/90 s off, and the library was prepared with 4–8 PCR cycles. The libraries were sequenced in a NovaSeq6000 instrument using a paired-end 2 × 150 bp protocol and aiming for 80 Gb/sample.

Data processing and EM-seq data analysis

We processed the raw sequencing data using the wg-blimp pipeline (v 0.10.0)[35] (Table S2). Originally designed as a pipeline for analyzing whole-genome-bisulfite-sequencing data, wg-blimp is also capable to handle enzymatic sequencing data, which shares the same raw data format but offers improved sequencing accuracy and reliability.[36] wg-blimp incorporates the well-established algorithms for each task within its pipeline. Namely, bwa-meth is used for alignment[37] against the GRCh38.p7 reference genome, MultiQC for quality control,[38] and MethylDackel (v 0.6.1) for methylation calling. As an integral component of wg-blimp, MethylDackel extracts per-base methylation metrics from the aligned and indexed BAM files alongside the corresponding reference genome. These metrics are formatted in the bedGraph standard, containing the counts of read pairs with evidence for methylated and unmethylated cytosines at the given genomic positions. Subsequently, camel (v 0.4.7) and metilene (v 0.2–8)[39] were employed for identifying differentially methylated regions (DMRs).

To visualize the distribution of DNA methylation across various functional regions, we utilized the annotation sources provided by wg-blimp for hg38 alignment, which includes gtf-annotation and masked repeats. These alignments were imported into R 4.2.1 using the "rtracklayer" (v 1.58.0),[40] while the identification of methylated regions of interest was performed using the "GenomicRanges" package (v 1.50.2).[41] Statistical analyses and graph plotting were performed using R packages, namely "stats" (v 4.2.1), "ggplot2" (v 3.3.6),[42] and "introdataviz" (v 0.0.0.9003).[43] For the analysis of methylation distribution, we considered only CpG sites with a minimum coverage of 5.

Principal component analysis (PCA) was conducted on the methylation values, which were obtained from the wg-blimp software using the tool MethylDackl. The PCA analysis was conducted on 2,521 CpG sites within the 50 known imprinting control regions (ICRs),[44] where all samples exhibited at least 5× coverage.

DMR analyses

DMR calling was conducted using metilene and camel within the wg-blimp software. The following criteria for DMR identification were applied: a minimum coverage of 5 per CpG loci, covering at least 5 CpG sites within a DMR, showing at least 20% methylation difference in the compared groups, and a maximum mean difference of ≤30% within each group for the CTR samples and ≤15% for the CZ samples to reduce the influence of genetic variability between individuals. For metilene, a threshold of $q < 0.05$ was set, whereas camel uses t statistics for verification.

Positive or negative enrichment of DMRs within specific genomic regions was assessed using permutation tests. The regions of interest were annotated using R 4.3.0 with the packages "stats" (v 4.3.0), "annotator"[45] and "TxDb.Hsapiens.UCSC.hg38.knownGene" (v 3.17.0), while the masked repeats were annotated using the annotation provided by wg-blimp. Permutation tests were performed using "regioneR"[46] and "GenomicRanges" using a Bonferroni correction ($\alpha = 0.00019$). Differences were quantified using z scores and p values provided by the permutation test with 10,000 iterations in "regioneR."

To evaluate the average expression and percentage of cells expressing the DMR-associated genes identified in this study, we used a previously published dataset.[16] Evaluation was performed using Seurat.[47,48] For better comparison to our dataset, we summarized the spermatocyte and spermatid cells from the single-cell RNA sequencing (scRNA-seq) dataset together. Germ-cell-type-specific genes for undifferentiated spermatogonia, differentiating spermatogonia, spermatocytes, and spermatids were extracted based on differential gene expression analysis using MAST.[49] Filtration criteria for germ-cell-type-specific genes were log fold change threshold of ≥0.5, false discovery rate (FDR)-corrected p value below 0.01, and expression in at least 25% of the cells of one comparison group.

Gene ontology (GO) enrichment analyses

"ChIPseeker"[50] was used to retrieve a comprehensive gene list of overlapping genes, gene promoters (transcriptional start sites [TSSs] ±1,000 bp) and flanking genes (putative regulatory sites, 5,000 bp) from our DMRs. These comprehensive DMR-associated gene lists were then analyzed for GO term enrichment for biological processes, molecular functions, and cellular components using "clusterProfiler"[51] and the enrichR database.[52] The p value was adjusted for multiple testing with Benjamini-Hochberg correction.

Motif analysis

To identify enriched known motifs of genes and transcription factor (TF) binding sites within the DMRs, we used Hypergeometric

Optimization of Motif Enrichment (HOMER) (v 4.11).[53] This tool was utilized with the default parameter of the fragments size of 200 bp and with the "-mask" parameter to use the repeat-masked sequences. Notably, HOMER uses regions with the same Guanine Cytosine-content distribution as control.

Retrieval of public datasets

We downloaded a dataset containing chromatin immunoprecipitation sequencing (ChIP-seq) data of enriched regions for retained histones (H3.3, H3K14ac, H3K27ac, H3K36me3, H3K4me1, and H3K9me3) in human sperm from Gene Expression Omnibus (GEO): GSE40195. The data were converted to GRCh38/hg19 using "rtracklayer."

Statistical analyses

Statistical analyses were performed as described in sections for data processing and EM-seq data analyses, DMR analysis, genomic annotation of the DMRs, and motif analysis.

Results

Primary spermatocytes exhibit genome-wide reduced DNA methylation levels

To investigate genome-wide DNA methylation changes during human spermatogenesis, we performed whole-genome EM-seq on isolated human male germ cells. We obtained undifferentiated spermatogonia (2C/MAGEA4^{+}/UTF1^{+}/DMRT1^{-}; Undiff), differentiating spermatogonia (2C/MAGEA4^{+}/UTF1^{-}/DMRT1^{+}; Diff), primary spermatocytes (double-diploid cells; 4C), and spermatids/sperm (haploid cells; 1C) from three men with normal spermatogenesis (CTR) (Figures 1A and S1A), which were separately used for EM-seq-based methylome analysis. We captured an average of 28,049,115 CpG sites per sample, including 26,861,322 CpGs with a minimum coverage of 5 reads, which were the ones considered for further analyses.

To guarantee that all germ cell fractions were free of somatic DNA, we compared the methylation of published sperm-soma DMRs[55] in our germ cell fractions with those published for sperm and blood samples.[54] We confirmed male germ-cell-specific methylation in the isolated cell fractions (Figure S1B). Interestingly, the methylation patterns of 50 known maternally and paternally methylated ICRs[44] were also similar to those found in sperm and had the same methylation pattern in all germ cell types (Figure 1B; Table S3). Analysis of global DNA methylation levels across spermatogenesis revealed comparably high average levels (>74%) of DNA methylation in undifferentiated spermatogonia, differentiating spermatogonia, and spermatids/sperm (Figure 1C), which is consistent with the characteristically high DNA methylation levels in male germ cells.[56] Intriguingly, the global DNA methylation levels in primary spermatocytes were significantly lower, with a mean of 66% (Figure 1C), compared to all other germ cell types. To elucidate whether the DNA hypomethylation in primary spermatocytes occurs randomly in the genome or is specific to particular genomic features, we analyzed the DNA methylation in gene bodies and 5,000 bp up- and downstream of the TSSs and transcriptional end sites (TESs). Our data showed that the decreased DNA methylation in primary spermatocytes, which was evident at TSS and TES, was particularly prominent in gene bodies (Figure 1D). Blood DNA displayed similar gene body methylation compared to spermatogonia and spermatids/sperm. However, the regions around and upstream of the TSS/downstream of the TES displayed higher methylation in blood than in all germ cell types evaluated. Deeper analysis of the different genomic compartments revealed a decline in DNA methylation in primary spermatocytes that occurred across untranslated regions (UTRs), transcripts, and RNA repeats. DNA methylation also declined at long terminal repeats (LTRs) and non-LTRs, including the TEs LINEs and SINEs, indicating a genome-wide demethylation in primary spermatocytes (Figure 1E). In contrast, there was no change in DNA methylation at centromeric and satellite regions, which overall display low DNA methylation levels in all germ cell types evaluated.

DMRs during spermatogenesis are enriched at SINE repeats

In order to identify regions that change their DNA methylation during spermatogenesis, we compared the methylomes of the different germ cell types (coverage ≥ 5, p value ≤ 0.05 (metilene), difference $\geq 20\%$). Applying stringent filtering, we identified a total of 16,000 DMRs throughout the different germ cell types (Figure 2A; Table S4). We found the fewest DMRs between undifferentiated and differentiating spermatogonia (64 DMRs, mean Δ methylation = 24%), indicating a high level of similarity between the methylomes of the two spermatogonial subpopulations. The most DMRs were detected between spermatogonia (undifferentiated and differentiating) and primary spermatocytes (5,212 and 5,487 DMRs, mean Δ methylation = 22% and 23%). When we compared the cell types corresponding to the least- and most-differentiated cell types, namely undifferentiated/differentiating spermatogonia and spermatids/sperm, we found the most extreme changes in DNA methylation (1,516 and 1,001 DMRs, mean Δ methylation = 41% and 36%). Analyses on the intersection of DMRs with genes or promoters revealed that 53%–70% are associated with genes and 3%–9% with a promoter (Figure 2B). To investigate the features of the DMRs, we analyzed their CpG enrichment and length. In comparison to other cell-type comparisons that had an average of 8 CpGs and a length of 340 bp, we found that DMRs obtained from the comparisons that included primary spermatocytes were the most enriched in CpGs (average 14 CpGs) and the longest (average 975 bp) (Figures 2C and 2D). As expected, there was a great overlap in the DMRs detected between the different types of spermatogonia and primary spermatocytes, in accordance with the high similarity between undifferentiated and differentiating spermatogonia (Figure 2E). We asked whether

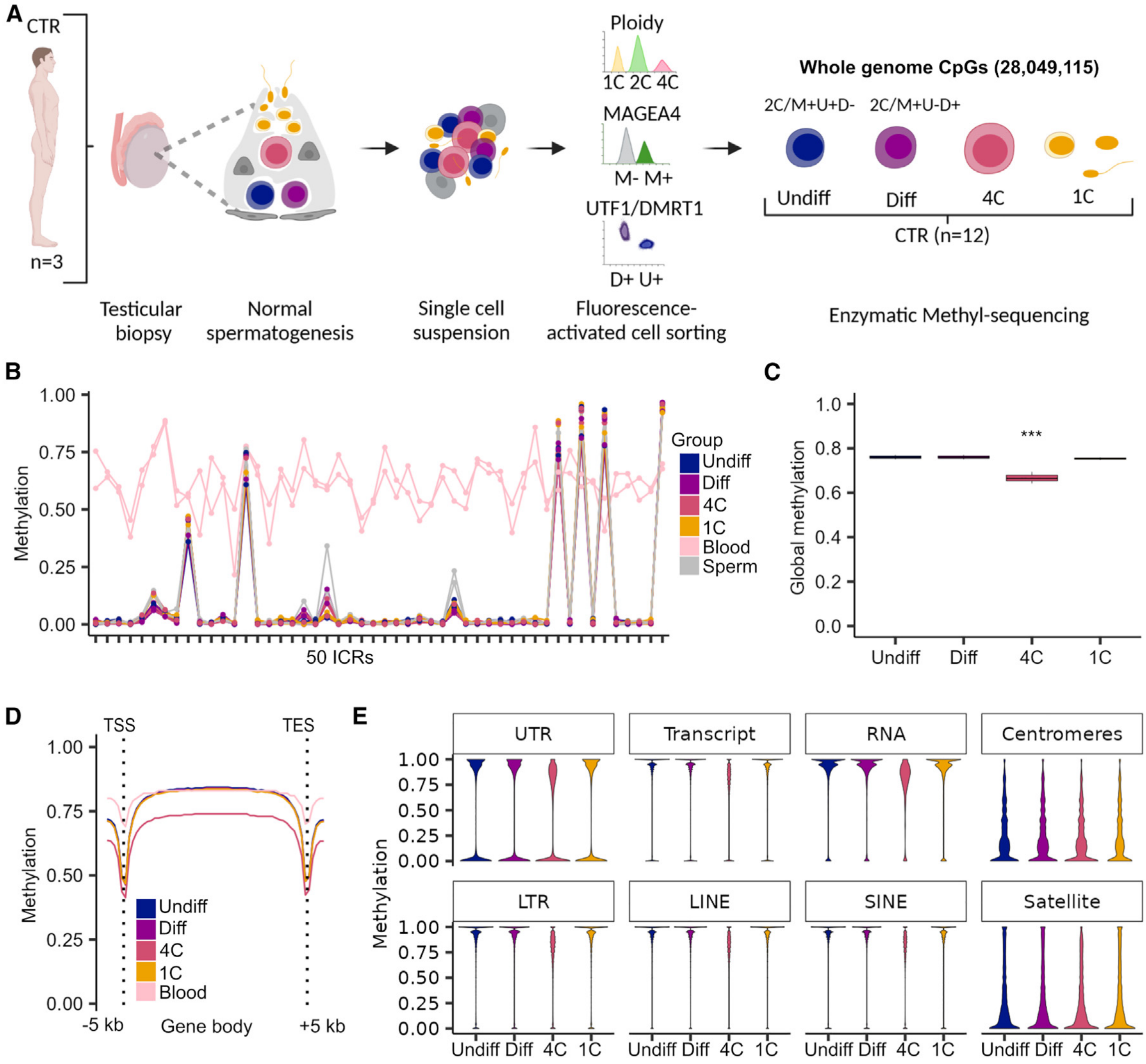

Figure 1. Primary spermatocytes exhibit genome-wide reduced DNA methylation levels
(A) Schematic illustration on the retrieval of whole-genome methylome data from germ cells of samples with normal spermatogenesis (control, CTR). CpGs refer to the mean CpG number captured by enzymatic methyl-sequencing in all germ cell fractions ($n = 12$).
(B) Line plot depicts the methylation in 50 imprinting control regions (ICRs) for each CTR sample and germ cell type compared to published blood and sperm samples.[54]
(C) Boxplots display the mean global DNA methylation levels. Statistical tests: ANOVA test followed by Tukey-HSD-test: *** <0.001 of 4C compared to all other germ cells. Data are represented as median (center line), upper/lower quartiles (box limits), 1.5× interquartile range (whiskers).
(D) Line plot shows mean methylation levels per group across gene bodies divided into 50 intervals (bins) and 5 kb upstream and downstream of the transcriptional start sites (TSSs) and transcriptional end sites (TESs).
(E) Violin plots represent methylated CpGs across different genomic compartments. Undiff, undifferentiated spermatogonia; Diff, differentiating spermatogonia; 4C, primary spermatocytes; 1C, spermatids/sperm.
(A) was created with BioRender.com. See also Figure S1.

certain chromosomes showed an enrichment in DMRs. To this end, we normalized the number of DMRs per chromosome size (bp)[57] and scaled for the number of DMRs per group comparison. We found that DMRs were similarly distributed across the different chromosomes in all comparisons, except for the 64 undifferentiated vs. differentiating spermatogonia DMRs, which showed a peak on the X chromosome (Figure 2F). Moreover, we sought to elucidate whether the DMRs are significantly enriched for certain genomic features. Intriguingly, enrichment analysis showed that particularly DMRs of the undifferentiated spermatogonia vs. spermatids/sperm comparison were significantly enriched (corrected p value ≤ 0.00019, Z score >0) at CpG shelves, 3′ UTRs, and exons, indicating changes in DNA methylation in these regions might be characteristic of the process of spermatogenesis (Figure 2G). We found reduced global DNA methylation levels of LINEs and SINEs exclusively in primary spermatocytes (Figure 1E). For all DMRs between the different germ cell types, we found a significant enrichment only at SINEs (Figure 2G), suggesting a role for DNA methylation changes in SINEs during spermatogenesis. In contrast, we

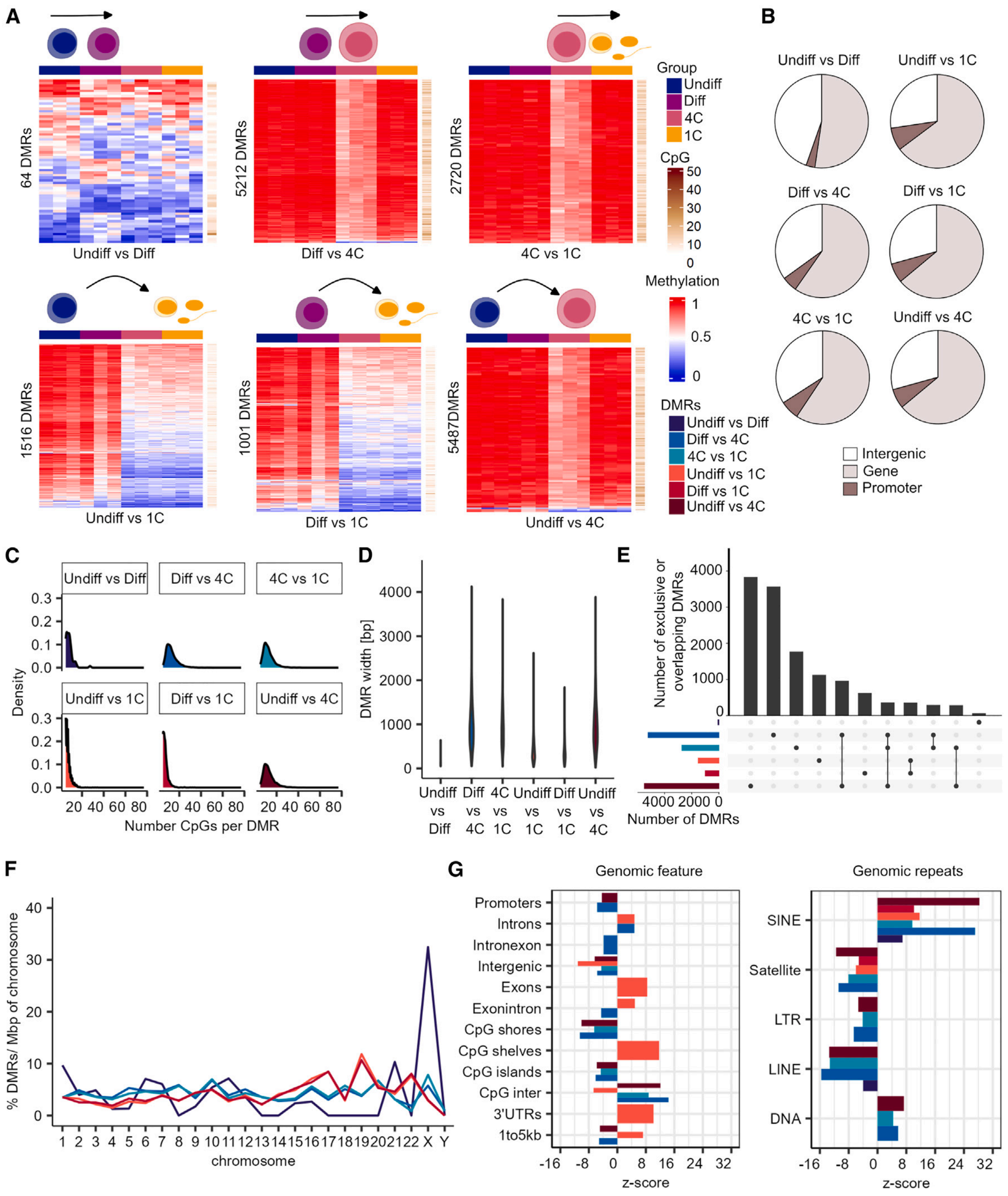

Figure 2. Differentially methylated regions during spermatogenesis are enriched at SINE repeats

(A) Heatmaps display methylation values and CpG numbers of the differentially methylated regions (DMRs) of all germ cell type comparisons.

(B) DMRs are associated with genes and promoters.

(C) Frequency of CpG number per DMR within the different group comparisons.

(D) Violin plot depicts distribution of the DMR width in each group comparison.

(E) Overlap (100%) of the DMRs among the different group comparisons. The exclusive DMRs are indicated by a dot, and the overlap of DMRs is shown by connecting nodes.

(F) Distribution of DMRs per chromosome scaled for chromosomal size (base pairs) and normalized by their total count within one group.

(G) Enrichment of DMRs for general genomic features and genomic repeats. Positive and negative enrichments are indicated by *Z* score. Displayed annotations, $p < 0.00019$ by permutation tests. Color coding of the group comparisons are depicted in (A). Undiff, undifferentiated spermatogonia; Diff, differentiating spermatogonia; 4C, primary spermatocytes; 1C, spermatids/sperm.

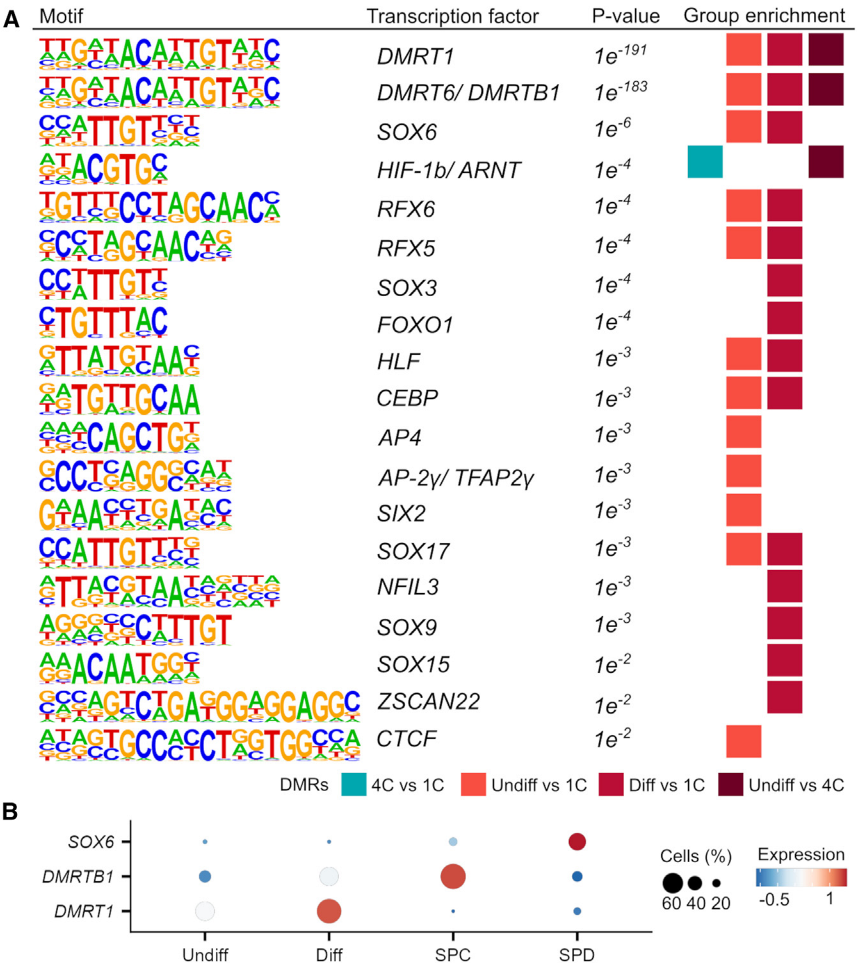

Figure 3. Hypomethylated regions in spermatids/sperm are enriched for specific TF binding sites

(A) Depicted are the enriched sequences of known motifs identified by HOMER. HOMER analysis was run for all DMR comparisons and significant results are displayed (p value <0.01, false discovery rate (FDR) < 0.05). Undiff, undifferentiated spermatogonia; Diff, differentiating spermatogonia; 4C, primary spermatocytes; 1C, spermatids/sperm.
(B) Dot plots show the average single-cell expression[16] of the top three transcription factors (TFs) with enriched motifs among the DMRs identified with HOMER. SPC, spermatocytes; SPD, spermatids.

found an underrepresentation, compared to what would be expected by chance (Z score <0), of promoters, CpG shores, and islands in DMRs of the differentiating spermatogonia vs. primary spermatocyte comparison, putatively indicating the methylation levels of these regions are maintained during the entry into meiosis. Interestingly, LINE elements were underrepresented in the linear DMR comparisons (i.e., Undiff vs. Diff, Diff vs. 4C, 4C vs. 1C) as well as between the undifferentiated spermatogonia vs. primary spermatocyte comparison, indicating a preferential DNA methylation retention at LINE repeat regions during spermatogenesis.

To unravel the putative regulatory role of DNA methylation changes in biological processes in the respective germ cell types, we investigated the nearby genes with a regulatory region within the identified DMRs. We found that the DMRs of all comparisons overlapped a putative regulatory region of 29–1,685 genes (Table S5). Within these genes, GO term analysis revealed an enrichment of distinct GO terms in biological processes and molecular functions, such as regulation of cation channel activity (Undiff vs. Diff) and GTPase regulatory activity (Undiff vs. 1C), (Figure S2) indicating changes in DNA methylation during spermatogenesis associates with specific cellular functions.

Hypomethylated regions in spermatids/sperm are enriched for TF binding sites

Local methylation changes can identify potential regulatory regions in developing germ cells.[58] To address whether regulatory TF binding sites undergo changes in DNA methylation during spermatogenesis, we analyzed the DMRs for their enrichment in TF binding motifs by applying HOMER analysis. This analysis revealed that particularly the DMRs of the spermatogonia (undifferentiated and differentiating) vs. spermatids/sperm comparisons were enriched for motifs recognized by TFs such as DMRT1, DMRT6/DMRTB1, and SOX6 (Figure 3A). To assess whether these TFs are expressed in the respective germ cell types, in which we found differential methylation of their motifs, we analyzed their expression during spermatogenesis in our published scRNA-seq dataset.[16] We found germ cell-type-specific expression of *DMRT1* (MIM: 602424), *DMRTB1* (MIM: 614805), and *SOX6* (MIM: 607257) in differentiating spermatogonia, spermatocytes, and spermatids, respectively (Figure 3B), suggesting the change in DNA methylation of the identified motifs is a feature of spermatogenesis.

Germ-cell-type-specific expression of the DMR-associated genes

Low or unmethylated regions frequently mark accessible chromatin regions.[59] As we found that the methylomes of spermatids/sperm, when compared to undifferentiated spermatogonia, have the highest number of hypomethylated DMRs and are enriched for TF binding sites, we hypothesized that the hypomethylated DMRs mark functionally important regions for spermatids/sperm. To this end, we evaluated whether the DMR-associated genes (overlapping a gene body) of the undifferentiated spermatogonia vs. spermatids/sperm comparison have specific

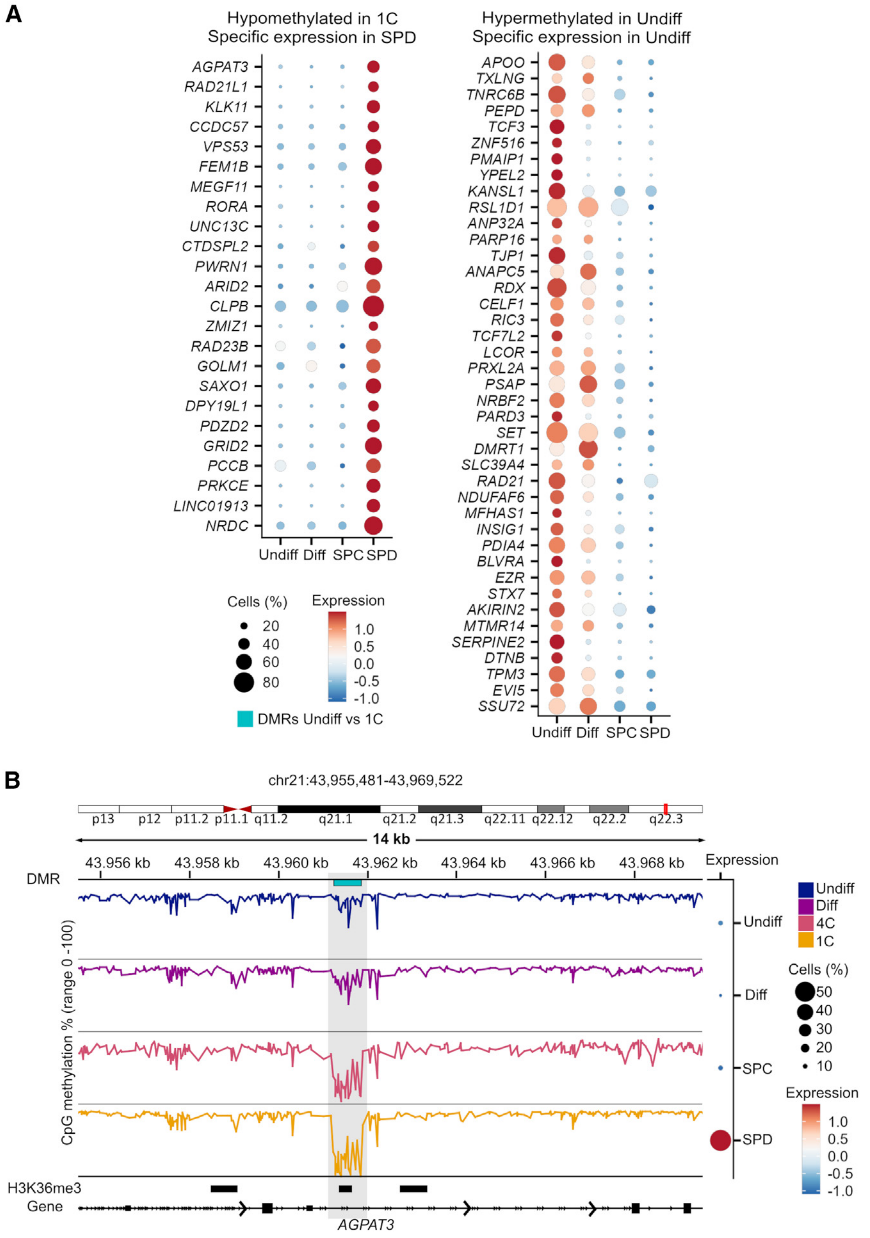

Figure 4. Hypomethylated regions in the spermatids/sperm methylomes mark spermatid-specific genes

(A) Single-cell expression[16] of hypomethylated DMR-associated genes with specific expression in spermatids and hypermethylated DMR-associated genes with specific expression in undifferentiated spermatogonia of the undifferentiated spermatogonia vs. 1C DMRs.

(B) Example of the DMR methylation within the *AGPAT3* locus that is hypermethylated in Undiff and Diff and hypomethylated in 4C and 1C and specifically expressed in SPD. H3K36me3 histone modification data (GSE40195) in human sperm is also shown. Undiff, undifferentiated spermatogonia; Diff, differentiating spermatogonia; 4C, primary spermatocytes; 1C, spermatids/sperm; SPC, spermatocytes; SPD, spermatids. See also Figure S3.

expression in spermatids. Indeed, we identified 24 highly spermatid-specific genes (e.g., *RAD21L1* [MIM: 619533], *KLK11* [MIM: 604434], *FEM1B* [MIM: 613539], *CLPB* [MIM: 616254], and *NRDC* [MIM: 602651]) among the hypomethylated DMRs (Figure 4A). Intriguingly, and in line with the association of gene expression and gene body methylation,[60] we found 41 spermatogonial-specific genes (e.g., *SERPINE2* [MIM: 177010], *BLVRA* [MIM: 109750], *TJP1* [MIM: 601009], *YPEL2* [MIM: 609723], and *TCF3* [MIM: 147141]) that have a hypermethylated DMR in their gene bodies in undifferentiated spermatogonia (Figure 4A). When we analyzed the DMR-associated genes of other group comparisons, we observed that the majority of genes expressed in a germ-cell-type-specific manner in our published scRNA-seq dataset[16] have hypermethylated DMRs (Figure S3A), and only a minority of them have hypomethylated DMRs in the respective germ cell types (Figure S3B).

Retained nucleosomes in human sperm were shown to be enriched at gene regulatory regions.[61,62] To examine whether the hypomethylated DMRs of the undifferentiated spermatogonia vs. spermatids/sperm comparison mark regulatory active regions in sperm, we analyzed the overlap of the hypomethylated DMRs with retained histone marks in sperm. Indeed, overlap analysis with publicly available datasets on human sperm histones (GSE40195) revealed that ∼17% of DMRs overlapped with a retained histone mark in sperm (Table S6). The active histone mark H3K36me3 overlaid 10% of the hypomethylated DMRs in spermatids/sperm. Although H3K36me3 is associated with gene body methylation to maintain gene expression stability,[63] we found H3K36me3 marked a hypomethylated region in *AGPAT3* (MIM: 614794), which is specifically expressed in spermatids (Figure 4B).

Disturbed spermatogenesis displays methylome changes at TEs and spermatogenesis genes

Aberrant DNA methylation was found in sperm of infertile men, particularly in imprinted genes.[26,64–68] To confidently associate changes in DNA methylation with male infertility, it is important to exclude effects of differential methylation, especially in imprinted genes, due to potential contamination with somatic DNA. Accordingly, it remains to be elucidated whether genome-wide changes in germ cells of infertile individuals occur. To this end, we analyzed methylomes of germ cells isolated from testicular

tissues of men diagnosed with CZ (Figure 5A). Ploidy analysis confirmed the characteristic cryptozoospermic phenotype,[16] consisting of a decreased proportion of spermatids/sperm in comparison to the CTR samples (Figure 5B). Based on cell numbers (Table S7), we were able to generate methylomes of undifferentiated spermatogonia, differentiating spermatogonia, and primary spermatocytes from these samples. Quality control[3,55] indicated the presence of somatic DNA in CZ-1 (Figures S4A and S4B), therefore we excluded all fractions from this subject from further analyses. The other two samples were free of somatic DNA and, except for one secondary ICR (MKRN3:TSS-DMR [MIM: 603856]) that showed an isolated increase in DNA methylation (average of 35%), showed no overall imprinting errors (Figure S4C; Table S8).

To examine whether regions exhibit differential methylation in cryptozoospermic subjects ($n = 2$) compared to CTR ($n = 3$), we analyzed potential DMRs between undifferentiated spermatogonia, differentiating spermatogonia, and primary spermatocytes. We applied the same DMR filtering as for the control germ cell DMRs (coverage ≥ 5, p value ≤ 0.05 [metilene], difference $\geq 20\%$), but due to the smaller sample size, we used stricter filtering parameters for the methylation range within the cryptozoospermic group (range $\leq 15\%$) to filter out DMRs potentially influenced by the genetic background. We found 107, 144, and 152 DMRs (mean Δ methylation = 31%–37%) in undifferentiated spermatogonia, differentiating spermatogonia, and primary spermatocytes between CTR and CZ samples (Figure 5C). We found few overlapping DMRs between all comparisons (Table S9). The DMRs were overall enriched in chromosomes 21 and X (Figure 5D) and significantly enriched in intergenic regions and at retroposons (Figure 5E), indicating that altered DNA methylation patterns in cryptozoospermic germ cells are predominantly affecting these regions. Particularly, hypomethylation of evolutionary young TEs have been associated with decreased fertility in mice.[13] Therefore, we examined whether we could find changes in DNA methylation in evolutionary younger (SVA D/F, L1Hs, and L1PA2-5) and older TEs (HERVH-int and L1M7) between control and cryptozoospermic subjects. We found that spermatogonia (undifferentiated and differentiating) and primary spermatocytes of cryptozoospermic men had a decrease in methylation in SVA D/F TEs (Figure 5F). When we analyzed the methylation levels of different TEs in each control and cryptozoospermic sample, we found that the hypomethylation in TEs of the cryptozoospermic group is driven by sample CZ-2, which showed hypomethylation not only in SVA D/F but also in SVA B/C, as well as L1HS, L1PA2, HERVH-int (Figure S5). Interestingly, SVA A TEs were hypermethylated in both cryptozoospermic samples and in all control samples.

In bulk germ cells of cryptozoospermic samples, changes in DNA methylation were associated with functionally relevant genomic regions.[3] Our analysis revealed that CTR/CZ DMRs are associated with putative regulatory regions of 29, 39, and 38 genes in undifferentiated spermatogonia, differentiating spermatogonia, and primary spermatocytes, respectively (Table S10), which were significantly enriched for genes involved in differentiation processes (e.g., cell morphogenesis involved in differentiation) (Figure S4D). Because we found 29%–40% of DMRs overlap a gene body (Figure S4E), we asked whether they associate with germ-cell-type-specific gene expression, potentially pointing to a relevant function for spermatogenesis. Gene expression analysis showed that 25%–33% DMR-associated genes overlap with genes encoding transcripts specifically expressed by spermatogonia (*AL157778.1*, *PCDH11X* [MIM: 300246], *EDA* [MIM: 300451], *ANKRD33B*, *AC087354.1*, *DACH2* [MIM: 300608], and *VPS37D* [MIM: 610039]), spermatocytes (*HSPBAP1* [MIM: 608263], *FOXK1* [MIM: 616302], *ARHGAP33* [MIM: 614902], *NBPF1* [MIM: 610501], and *SLCO3A1* [MIM: 612435]), or spermatids (*CCDC200*, *AL589935.1*, *ADARB2* [MIM: 602065], and *SNHG27*) (Figure S4F). In summary, although we could not detect overall changes at ICRs, we found that cryptozoospermic germ cells exhibit changes in DNA methylation particularly occurring at evolutionarily younger TEs of the SAV family and at spermatogenic genes.

Discussion

The establishment of the germ cell methylome extends beyond prenatal development.[1–5] In this study, we used whole-methylome sequencing of germ cells to identify changes in DNA methylation occurring during human spermatogenesis. This included demethylation in primary spermatocytes followed by remethylation of specific regions, resulting in a unique spermatid-/sperm-specific DNA methylation pattern (Figure S6). Furthermore, we identified changes in the DNA methylation of germ cells from infertile men particularly affecting intergenic regions and TEs. We found that, during spermatogonial differentiation, only a few regions, predominantly located on the X chromosome, undergo changes in DNA methylation. Although the X chromosome is enriched for genes expressed by spermatogonia,[69] the 64 DMRs were not associated with genes involved in spermatogonial function or entry into meiosis. Interestingly, one of the DMR-associated genes for this comparison was *HDAC1* (MIM: 601241), a histone deacetylase, which has been implicated in chromatin remodeling and gene regulation during spermatogenesis.[70] Moreover, this gene is pivotal for correct DNA methylation patterns in mouse oocytes, including imprinting establishment.[71] It remains to be demonstrated whether DNA methylation is involved in the regulation of *HDAC1* expression and if this deacetylase is involved in the process of genome-wide DNA methylation remodeling during human spermatogenesis. Previous studies have reported that the male germline genome becomes hypomethylated during meiosis. They hypothesized that this decline in methylation is due to a

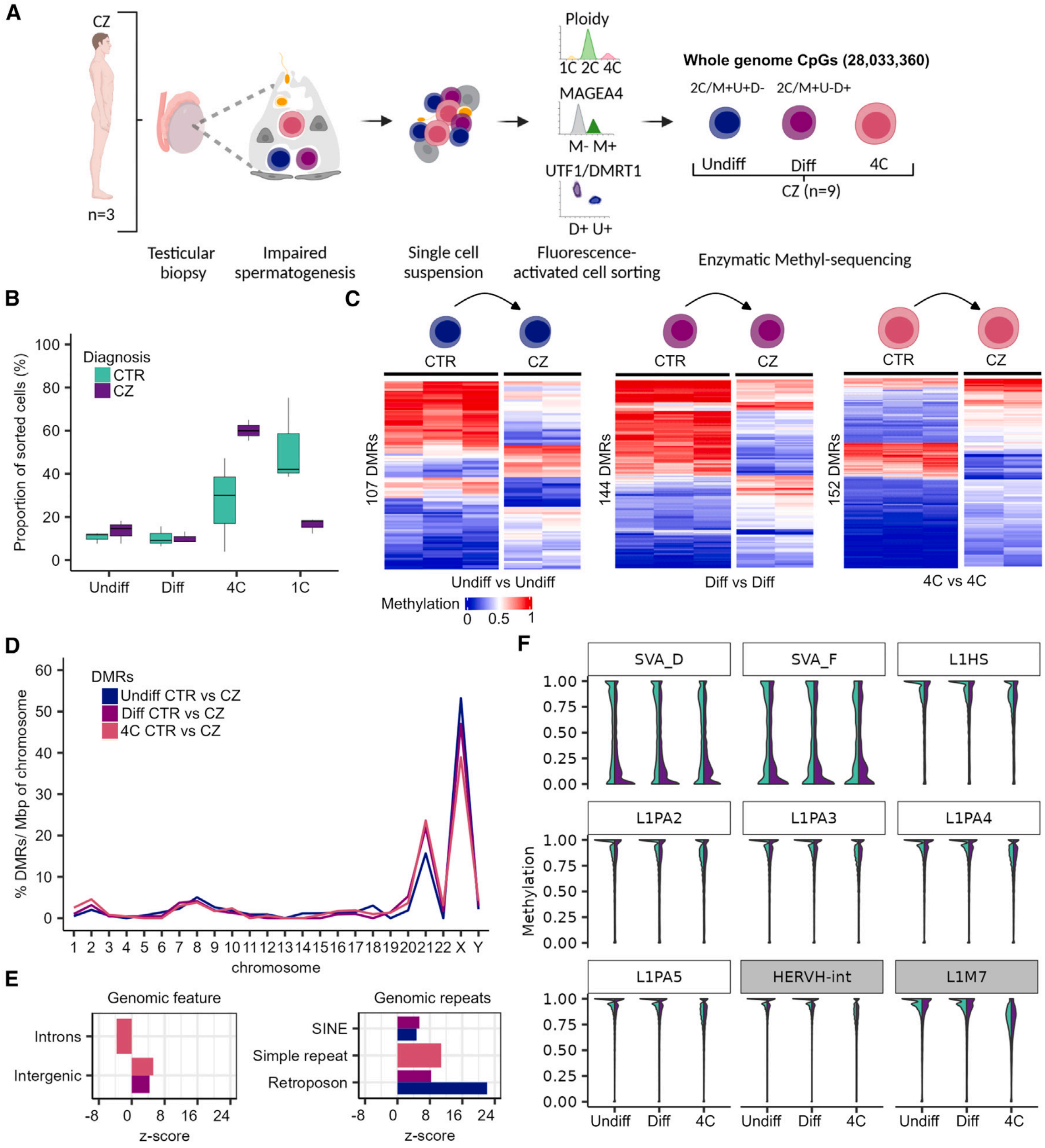

Figure 5. Disturbed spermatogenesis displays methylome changes at TEs and spermatogenesis genes
(A) Schematic illustration on the retrieval of whole-genome methylome data of germ cells from samples with disturbed spermatogenesis (CZ, cryptozoospermia).
(B) Boxplots show the proportion of cell types among the sorted cells in the CTR and CZ subjects. Data are represented as median (center line), upper/lower quartiles (box limits), 1.5× interquartile range (whiskers).
(C) Heatmaps display methylation values of the differentially methylated regions (DMRs) between CTR and CZ of the same cell type (Undiff vs. Undiff, Diff vs. Diff, and 4C vs. 4C).
(D) Distribution of the CTR/CZ DMRs per chromosome scaled for chromosomal size (base pairs) and normalized by their total count within one group.
(E) Enrichment of CTR/CZ DMRs for functional general genomic regions and genomic repeats. Positive and negative enrichments are indicated by *Z* score. Displayed annotations, $p < 0.00019$ by permutation tests.
(F) Violin plots showing the CpG methylation of evolutionary younger (white boxes: L1Hs, L1PA2-5, and SVA_D/F) and older (gray boxes: HERVH-int and L1M7) TEs in CTR and CZ germ cells.
Color coding of the group comparisons are depicted in (B). Undiff, undifferentiated spermatogonia; Diff, differentiating spermatogonia; 4C, primary spermatocytes; 1C, spermatids/sperm. (A) created with BioRender.com.
See also Figures S4 and S5.

delay in DNA methylation maintenance[6] and is associated with meiotic recombination.[5] Here, we show that the demethylation occurring in human male meiosis affects gene bodies and genomic repeats similarly but does not affect centromeres and satellite regions. Our data support the hypothesis of a replication-dependent passive DNA demethylation in early meiosis resulting in a hypomethylated genome in spermatocytes.[5,6] This phase is followed by an increase in average DNA methylation to levels similar to those prior to meiosis. However, careful examination of regions showing differential methylation demonstrates that discrete regions remain protected from re-methylation in spermatids/sperm. This indicates that genome-wide re-methylation in spermatids/sperm is not an outcome of DNA maintenance machinery restoring CpG methylation after meiosis, but instead evidences the establishment of a highly specific DNA methylation pattern in spermatids/sperm.

Our findings lead us to hypothesize that a functional relationship exists between the establishment of a spermatid-/sperm-specific methylome and gene expression during spermatogenesis. This notion was reinforced by our finding that hypomethylated regions in spermatids/sperm retain active histones in sperm. These regions correspond to genes specifically expressed by spermatids. Our hypothesis is further corroborated by analogous results in rodents, which demonstrate that the spermatid methylome undergoes differential methylation during spermiogenesis and is enriched for regulatory binding sites when compared to spermatogonial stem cells.[4,58]

During normal human spermatogenesis, we showed that primary spermatocytes were the only germ cell type displaying a global decrease in DNA methylation at LTRs, LINEs, and SINEs. The silencing of TEs is crucial for maintaining genome integrity and usually achieved by epigenetic modifications, including repressive histone modifications and DNA methylation.[17] In line with this, our data showed that LINEs are significantly underrepresented among the regions with differential methylation, indicating that the methylation status of LINEs is protected in adult male germ cells. In contrast, we found that DNA methylation at SINEs significantly changes in all germ cell types during spermatogenesis, indicating that these regions are not under the same tight regulation as LINEs. Consistent with our data on genome-wide DNA methylation changes at SINE elements, a recent study found varying levels of SINE methylation in sperm, specifically at promoters of spermatogenic genes.[72] Considering our data and the role of TEs in the regulation of genes,[17,73] we hypothesize that SINE methylation might play a regulatory role in human spermatogenesis.

Mice deficient for genes belonging to the DNA methylation machinery display male infertility or sterility.[10–14] In humans, the hypothesis that male infertility is associated with aberrations in DNA methylation was first explored by numerous studies reporting epimutations in the sperm of infertile men,[74] a notion that was somewhat challenged by our findings that somatic DNA artifacts and gene variants may have confounded previous studies.[55] However, more recently we identified consistent DNA methylation changes in testicular germ cells, but not in sperm, of infertile men and hypothesized that DNA methylation abnormalities during spermatogenesis might in fact be associated with disturbed spermatogenesis, specifically with CZ.[3] Here, we have expanded and tested this hypothesis and identified clear changes in DNA methylation at specific steps of spermatogenesis in germ cells of infertile men with CZ. Intriguingly, although these changes were germ cell type specific, the affected regions were consistently enriched in TEs and predominantly localized on chromosomes 21 and X, chromosomes associated with monogenic disorders, and male germ cell differentiation.[75–77] In one of the cryptozoospermic samples, we specifically found hypomethylated SVA elements together with hypomethylated L1HS, which is required to express SVA elements.[78] This finding shows similarities with a study demonstrating an association between hypomethylated young TEs and male infertility in mice.[13] DNA methylation is one of the main repressors of TE expression.[17–20] Therefore, we hypothesize that the protection of evolutionary young TEs of the SVA family is disrupted in this individual's germline leading to genomic instability due to the expression of SVAs and a phenotype of disturbed spermatogenesis. However, limited material prevents us from substantiating this at transcriptional level. Our findings on aberrant TE methylation in germ cells of men with CZ are, nevertheless, supported by studies showing that loss of DNA methylation at TEs results in sterility in mice.[12,21] Failure in TE silencing affects germline gene expression in mice[21] and, especially during meiosis, can lead to meiotic arrest.[22,23,79] In cryptozoospermic subjects, some seminiferous tubules show complete spermatogenesis while others display spermatogenic arrest.[16] This heterogeneous phenotype could be caused by epimutations only present in cells derived from affected spermatogonial clones[3] in contrast to genetic variations that usually lead to complete phenotype penetrance. In line with this, we found that numerous infertility-related DMR-associated genes are expressed during spermatogenesis (e.g., *VPS37D*, *FAM9B* [MIM: 300478], *SLCO3A1*, and *CCDC200*). In our previous study,[3] we found that DMR-associated genes in CZ-derived testicular germ cells were expressed during or post-meiosis. This might indicate that the presence of epimutations at earlier germ cell stages leads to an impairment in spermatogenesis. However, it remains unsolved how these epimutations contribute to the differences in spermatogenesis across tubules in cryptozoospermic individuals.

In conclusion, our findings provide an in-depth view of genome-wide DNA methylation changes during human spermatogenesis, highlighting the role of DNA methylation, particularly at TEs, during this process, and indicating a potential role for altered TE methylation in the etiology of human male infertility.

Data and code availability

EM-seq data have been deposited in the European Genome-Phenome Archive: EGAS00001007449. A previously published dataset used in this manuscript is available from GEO: GSE153947. Previously published ChIP-seq data is available from GEO: GSE40195.

The code generated for this study is available from GitHub: https://github.com/VerenaDietrich/Spermatogenesis-Methylome.

Supplemental information

Supplemental information can be found online at https://doi.org/10.1016/j.ajhg.2024.04.017.

Acknowledgments

We thank the Cologne Center for preparing the sequencing libraries and performing the sequencing. We appreciate the excellent technical support in subject material collection, histological evaluation of the testicular tissues, and hormone measurement by Nicole Terwort, Sabine Forsthoff, Heidi Kersebom, Elke Kößer and Elisabeth Lahrmann, as well as help from the andrology lab (CeRA). Panels for Figures 1A and 5A and the graphical abstract were created with BioRender.com.

This study was carried out within the frame of the Deutsche Forschungsgemeinschaft (DFG, German Research Foundation)-sponsored Clinical Research Unit 'Male Germ Cells' (CRU326, project numbers LA 4064/3-2 and NE 2190/3-2 to N.N. and S.L., respectively), a DFG grant 4064/4-1 (to S.L.), and an Innovative Medical Research (IMF) of the Medical Faculty of the University of Münster grant (to S.D.P.).

Author contributions

Study design, N.N. and S.L.; conceptualization, L.M.S.-K., V.D., N.N., and S.L.; subject counseling, J.-F.C. and S.K.; data curation, L.M.S.-K, J.B., M.S., J.-F.C., and S.K.; formal analysis, L.M.S.-K., V.D., S.D.P., and M.S.; investigation, L.M.S.-K., V.D., S.D.P., J.M.V., N.N., and S.L.; supervision, S. SSchlatt, S. Sandmann, J.V., S.K., J.M.V., N.N., and S.L.; funding acquisition, S.D.P., J.M.V., N.N., and S.L.; writing — original draft, L.M.S.-K., V.D., N.N., and S.L.; writing — review & editing, all authors.

All authors approved the final version of the manuscript.

Declaration of interests

The authors declare no competing interests.

Received: November 28, 2023
Accepted: April 23, 2024
Published: May 16, 2024

Web resources

annotatR, https://bioconductor.org/packages/release/bioc/html/annotatr.html.
BWA-meth, https://github.com/brentp/bwa-meth
camel, https://eldorado.tu-dortmund.de/handle/2003/37940.
ChIPseeker, https://bioconductor.org/packages/release/bioc/html/ChIPseeker.html
clusterProfiler, https://bioconductor.org/packages/release/bioc/html/clusterProfiler.html
enrichR, https://maayanlab.cloud/Enrichr/
GenomicRanges, https://bioconductor.org/packages/release/bioc/html/GenomicRanges.html
ggplot2, https://ggplot2.tidyverse.org/
HOMER, http://homer.ucsd.edu/homer/
introdataviz, https://github.com/PsyTeachR/introdataviz.
MAST, https://bioconductor.org/packages/release/bioc/html/MAST.html.
MethylDackel https://github.com/dpryan79/methyldackel.
Metilene, http://legacy.bioinf.uni-leipzig.de/Software/metilene/
MultiQC, https://multiqc.info/
regioneR, https://bioconductor.org/packages/release/bioc/html/regioneR.html.
R Project, https://www.r-project.org/
rtracklayer, https://www.bioconductor.org/packages/release/bioc/html/rtracklayer.html.
Seurat, https://satijalab.org/seurat/
TxDb.Hsapiens.UCSC.hg38.knownGene, https://bioconductor.org/packages/release/data/annotation/html/TxDb.Hsapiens.UCSC.hg38.knownGene.html
wg-blimp, https://github.com/MarWoes/wg-blimp.

References

1. Oakes, C.C., La Salle, S., Smiraglia, D.J., Robaire, B., and Trasler, J.M. (2007). Developmental acquisition of genome-wide DNA methylation occurs prior to meiosis in male germ cells. Dev. Biol. *307*, 368–379. https://doi.org/10.1016/j.ydbio.2007.05.002.
2. Langenstroth-Röwer, D., Gromoll, J., Wistuba, J., Tröndle, I., Laurentino, S., Schlatt, S., and Neuhaus, N. (2017). De novo methylation in male germ cells of the common marmoset monkey occurs during postnatal development and is maintained in vitro. Epigenetics *12*, 527–539. https://doi.org/10.1080/15592294.2016.1248007.
3. Di Persio, S., Leitão, E., Wöste, M., Tekath, T., Cremers, J.-F., Dugas, M., Li, X., Meyer zu Hörste, G., Kliesch, S., Laurentino, S., et al. (2021). Whole-genome methylation analysis of testicular germ cells from cryptozoospermic men points to recurrent and functionally relevant DNA methylation changes. Clin. Epigenet. *13*, 160. https://doi.org/10.1186/s13148-021-01144-z.
4. El Omri-Charai, R., Gilbert, I., Prunier, J., Desmarais, R., Ghinet, M.G., Robert, C., Boissonneault, G., and Delbes, G. (2023). DNA methylation dynamic in male rat germ cells during gametogenesis. Development *150*, dev201606. https://doi.org/10.1242/dev.201606.
5. Huang, Y., Li, L., An, G., Yang, X., Cui, M., Song, X., Lin, J., Zhang, X., Yao, Z., Wan, C., et al. (2023). Single-cell multi-omics sequencing of human spermatogenesis reveals a DNA demethylation event associated with male meiotic recombination. Nat. Cell Biol. *25*, 1520–1534. https://doi.org/10.1038/s41556-023-01232-7.
6. Gaysinskaya, V., Miller, B.F., De Luca, C., van der Heijden, G.W., Hansen, K.D., and Bortvin, A. (2018). Transient reduction of DNA methylation at the onset of meiosis in male mice. Epigenet. Chromatin *11*, 15. https://doi.org/10.1186/s13072-018-0186-0.

7. Klemm, S.L., Shipony, Z., and Greenleaf, W.J. (2019). Chromatin accessibility and the regulatory epigenome. Nat. Rev. Genet. *20*, 207–220. https://doi.org/10.1038/s41576-018-0089-8.
8. Markenscoff-Papadimitriou, E., Whalen, S., Przytycki, P., Thomas, R., Binyameen, F., Nowakowski, T.J., Kriegstein, A.R., Sanders, S.J., State, M.W., Pollard, K.S., and Rubenstein, J.L. (2020). A Chromatin Accessibility Atlas of the Developing Human Telencephalon. Cell *182*, 754–769.e18. https://doi.org/10.1016/j.cell.2020.06.002.
9. Izzo, F., Lee, S.C., Poran, A., Chaligne, R., Gaiti, F., Gross, B., Murali, R.R., Deochand, S.D., Ang, C., Jones, P.W., et al. (2020). DNA methylation disruption reshapes the hematopoietic differentiation landscape. Nat. Genet. *52*, 378–387. https://doi.org/10.1038/s41588-020-0595-4.
10. Bourc'his, D., Xu, G.-L., Lin, C.-S., Bollman, B., and Bestor, T.H. (2001). Dnmt3L and the Establishment of Maternal Genomic Imprints. Science *294*, 2536–2539. https://doi.org/10.1126/science.1065848.
11. Zamudio, N., Barau, J., Teissandier, A., Walter, M., Borsos, M., Servant, N., and Bourc'his, D. (2015). DNA methylation restrains transposons from adopting a chromatin signature permissive for meiotic recombination. Genes Dev. *29*, 1256–1270. https://doi.org/10.1101/gad.257840.114.
12. Barau, J., Teissandier, A., Zamudio, N., Roy, S., Nalesso, V., Hérault, Y., Guillou, F., and Bourc'his, D. (2016). The DNA methyltransferase DNMT3C protects male germ cells from transposon activity. Science *354*, 909–912. https://doi.org/10.1126/science.aah5143.
13. Karahan, G., Chan, D., Shirane, K., McClatchie, T., Janssen, S., Baltz, J.M., Lorincz, M., and Trasler, J. (2021). Paternal MTHFR deficiency leads to hypomethylation of young retrotransposons and reproductive decline across two successive generations. Development *148*, dev199492. https://doi.org/10.1242/dev.199492.
14. Dura, M., Teissandier, A., Armand, M., Barau, J., Lapoujade, C., Fouchet, P., Bonneville, L., Schulz, M., Weber, M., Baudrin, L.G., et al. (2022). DNMT3A-dependent DNA methylation is required for spermatogonial stem cells to commit to spermatogenesis. Nat. Genet. *54*, 469–480. https://doi.org/10.1038/s41588-022-01040-z.
15. Tüttelmann, F., Ruckert, C., and Röpke, A. (2018). Disorders of spermatogenesis: Perspectives for novel genetic diagnostics after 20 years of unchanged routine. medgen *30*, 12–20. https://doi.org/10.1007/s11825-018-0181-7.
16. Di Persio, S., Tekath, T., Siebert-Kuss, L.M., Cremers, J.-F., Wistuba, J., Li, X., Meyer zu Hörste, G., Drexler, H.C.A., Wyrwoll, M.J., Tüttelmann, F., et al. (2021). Single-cell RNA-seq unravels alterations of the human spermatogonial stem cell compartment in patients with impaired spermatogenesis. Cell Rep. Med. *2*, 100395. https://doi.org/10.1016/j.xcrm.2021.100395.
17. Zamudio, N., and Bourc'his, D. (2010). Transposable elements in the mammalian germline: a comfortable niche or a deadly trap? Heredity *105*, 92–104. https://doi.org/10.1038/hdy.2010.53.
18. Schaefer, C.B., Ooi, S.K.T., Bestor, T.H., and Bourc'his, D. (2007). Epigenetic Decisions in Mammalian Germ Cells. Science *316*, 398–399. https://doi.org/10.1126/science.1137544.
19. Vaissiere, T., Sawan, C., and Herceg, Z. (2008). Epigenetic interplay between histone modifications and DNA methylation in gene silencing. Mutat. Res. Rev. Mutat. Res. *659*, 40–48. https://doi.org/10.1016/j.mrrev.2008.02.004.
20. Dong, J., Wang, X., Cao, C., Wen, Y., Sakashita, A., Chen, S., Zhang, J., Zhang, Y., Zhou, L., Luo, M., et al. (2019). UHRF1 suppresses retrotransposons and cooperates with PRMT5 and PIWI proteins in male germ cells. Nat. Commun. *10*, 4705. https://doi.org/10.1038/s41467-019-12455-4.
21. Vasiliauskaitė, L., Berrens, R.V., Ivanova, I., Carrieri, C., Reik, W., Enright, A.J., and O'Carroll, D. (2018). Defective germline reprogramming rewires the spermatogonial transcriptome. Nat. Struct. Mol. Biol. *25*, 394–404. https://doi.org/10.1038/s41594-018-0058-0.
22. Bourc'his, D., and Bestor, T.H. (2004). Meiotic catastrophe and retrotransposon reactivation in male germ cells lacking Dnmt3L. Nature *431*, 96–99. https://doi.org/10.1038/nature02886.
23. Carmell, M.A., Girard, A., van de Kant, H.J.G., Bourc'his, D., Bestor, T.H., de Rooij, D.G., and Hannon, G.J. (2007). MIWI2 Is Essential for Spermatogenesis and Repression of Transposons in the Mouse Male Germline. Dev. Cell *12*, 503–514. https://doi.org/10.1016/j.devcel.2007.03.001.
24. Aravin, A.A., Sachidanandam, R., Girard, A., Fejes-Toth, K., and Hannon, G.J. (2007). Developmentally Regulated piRNA Clusters Implicate MILI in Transposon Control. Science *316*, 744–747. https://doi.org/10.1126/science.1142612.
25. Heyn, H., Ferreira, H.J., Bassas, L., Bonache, S., Sayols, S., Sandoval, J., Esteller, M., and Larriba, S. (2012). Epigenetic Disruption of the PIWI Pathway in Human Spermatogenic Disorders. PLoS One *7*, e47892. https://doi.org/10.1371/journal.pone.0047892.
26. Urdinguio, R.G., Bayón, G.F., Dmitrijeva, M., Toraño, E.G., Bravo, C., Fraga, M.F., Bassas, L., Larriba, S., and Fernández, A.F. (2015). Aberrant DNA methylation patterns of spermatozoa in men with unexplained infertility. Hum. Reprod. *30*, 1014–1028. https://doi.org/10.1093/humrep/dev053.
27. Fukuda, K., Makino, Y., Kaneko, S., Shimura, C., Okada, Y., Ichiyanagi, K., and Shinkai, Y. (2022). Potential role of KRAB-ZFP binding and transcriptional states on DNA methylation of retroelements in human male germ cells. Elife *11*, e76822. https://doi.org/10.7554/eLife.76822.
28. Seisenberger, S., Andrews, S., Krueger, F., Arand, J., Walter, J., Santos, F., Popp, C., Thienpont, B., Dean, W., and Reik, W. (2012). The Dynamics of Genome-wide DNA Methylation Reprogramming in Mouse Primordial Germ Cells. Mol. Cell. *48*, 849–862. https://doi.org/10.1016/j.molcel.2012.11.001.
29. Kobayashi, H., Sakurai, T., Imai, M., Takahashi, N., Fukuda, A., Yayoi, O., Sato, S., Nakabayashi, K., Hata, K., Sotomaru, Y., et al. (2012). Contribution of Intragenic DNA Methylation in Mouse Gametic DNA Methylomes to Establish Oocyte-Specific Heritable Marks. PLoS Genet. *8*, e1002440. https://doi.org/10.1371/journal.pgen.1002440.
30. Gkountela, S., Zhang, K.X., Shafiq, T.A., Liao, W.-W., Hargan-Calvopiña, J., Chen, P.-Y., and Clark, A.T. (2015). DNA Demethylation Dynamics in the Human Prenatal Germline. Cell *161*, 1425–1436. https://doi.org/10.1016/j.cell.2015.05.012.
31. Guo, F., Yan, L., Guo, H., Li, L., Hu, B., Zhao, Y., Yong, J., Hu, Y., Wang, X., Wei, Y., et al. (2015). The Transcriptome and DNA Methylome Landscapes of Human Primordial Germ Cells. Cell *161*, 1437–1452. https://doi.org/10.1016/j.cell.2015.05.015.
32. World Health Organization (2021). WHO Laboratory Manual for the Examination and Processing of Human Semen (World Health Organization).

33. Bergmann, M., and Kliesch, S. (2010). Testicular biopsy and histology. In Andrology, E. Nieschlag, H.M. Behre, and S. Nieschlag, eds. (Springer).
34. Siebert-Kuss, L.M., Krenz, H., Tekath, T., Wöste, M., Di Persio, S., Terwort, N., Wyrwoll, M.J., Cremers, J.-F., Wistuba, J., Dugas, M., et al. (2023). Transcriptome analyses in infertile men reveal germ cell–specific expression and splicing patterns. Life Sci. Alliance *6*, e202201633. https://doi.org/10.26508/lsa.202201633.
35. Wöste, M., Leitão, E., Laurentino, S., Horsthemke, B., Rahmann, S., and Schröder, C. (2020). wg-blimp: an end-to-end analysis pipeline for whole genome bisulfite sequencing data. BMC Bioinf. *21*, 169. https://doi.org/10.1186/s12859-020-3470-5.
36. Feng, S., Zhong, Z., Wang, M., and Jacobsen, S.E. (2020). Efficient and accurate determination of genome-wide DNA methylation patterns in Arabidopsis thaliana with enzymatic methyl sequencing. Epigenet. Chromatin *13*, 42. https://doi.org/10.1186/s13072-020-00361-9.
37. Pedersen, B.S., Eyring, K., De, S., Yang, I.V., and Schwartz, D.A. (2014). Fast and accurate alignment of long bisulfite-seq reads. Preprint at arXiv. https://doi.org/10.48550/arXiv.1401.1129.
38. Ewels, P., Magnusson, M., Lundin, S., and Käller, M. (2016). MultiQC: summarize analysis results for multiple tools and samples in a single report. Bioinformatics *32*, 3047–3048. https://doi.org/10.1093/bioinformatics/btw354.
39. Jühling, F., Kretzmer, H., Bernhart, S.H., Otto, C., Stadler, P.F., and Hoffmann, S. (2016). metilene: fast and sensitive calling of differentially methylated regions from bisulfite sequencing data. Genome Res. *26*, 256–262. https://doi.org/10.1101/gr.196394.115.
40. Lawrence, M., Gentleman, R., and Carey, V. (2009). rtracklayer: an R package for interfacing with genome browsers. Bioinformatics *25*, 1841–1842. https://doi.org/10.1093/bioinformatics/btp328.
41. Lawrence, M., Huber, W., Pagès, H., Aboyoun, P., Carlson, M., Gentleman, R., Morgan, M.T., and Carey, V.J. (2013). Software for Computing and Annotating Genomic Ranges. PLoS Comput. Biol. *9*, e1003118. https://doi.org/10.1371/journal.pcbi.1003118.
42. Wickham, H. (2016). ggplot2: Elegant Graphics for Data Analysis (New York: Springer-Verlag).
43. Nordmann, E., McAleer, P., Toivo, W., Paterson, H., and DeBruine, L.M. (2022). Data Visualization Using R for Researchers Who Do Not Use R. Advances in Methods and Practices in Psychological Science *5*, 251524592210746. https://doi.org/10.1177/25152459221074654.
44. Monk, D., Morales, J., den Dunnen, J.T., Russo, S., Court, F., Prawitt, D., Eggermann, T., Beygo, J., Buiting, K., Tümer, Z.; and Nomenclature group of the European Network for Human Congenital Imprinting Disorders (2018). Recommendations for a nomenclature system for reporting methylation aberrations in imprinted domains. Epigenetics *13*, 117–121. https://doi.org/10.1080/15592294.2016.1264561.
45. Cavalcante, R.G., and Sartor, M.A. (2017). annotatr: genomic regions in context. Bioinformatics *33*, 2381–2383. https://doi.org/10.1093/bioinformatics/btx183.
46. Gel, B., Díez-Villanueva, A., Serra, E., Buschbeck, M., Peinado, M.A., and Malinverni, R. (2016). regioneR: an R/Bioconductor package for the association analysis of genomic regions based on permutation tests. Bioinformatics *32*, 289–291. https://doi.org/10.1093/bioinformatics/btv562.
47. Stuart, T., Butler, A., Hoffman, P., Hafemeister, C., Papalexi, E., Mauck, W.M., Hao, Y., Stoeckius, M., Smibert, P., and Satija, R. (2019). Comprehensive integration of single-cell data. Cell *177*, 1888–1902.e21. https://doi.org/10.1016/j.cell.2019.05.031.
48. Hao, Y., Hao, S., Andersen-Nissen, E., Mauck, W.M., Zheng, S., Butler, A., Lee, M.J., Wilk, A.J., Darby, C., Zager, M., et al. (2021). Integrated analysis of multimodal single-cell data. Cell *184*, 3573–3587.e29. https://doi.org/10.1016/j.cell.2021.04.048.
49. Finak, G., McDavid, A., Yajima, M., Deng, J., Gersuk, V., Shalek, A.K., Slichter, C.K., Miller, H.W., McElrath, M.J., Prlic, M., et al. (2015). MAST: a flexible statistical framework for assessing transcriptional changes and characterizing heterogeneity in single-cell RNA sequencing data. Genome Biol. *16*, 278. https://doi.org/10.1186/s13059-015-0844-5.
50. Yu, G., Wang, L.-G., and He, Q.-Y. (2015). ChIPseeker: an R/Bioconductor package for ChIP peak annotation, comparison and visualization. Bioinformatics *31*, 2382–2383. https://doi.org/10.1093/bioinformatics/btv145.
51. Wu, T., Liu, W., Huang, S., Chen, J., He, F., Wang, H., Zheng, X., Li, Z., Zhang, H., Zha, Z., et al. (2021). clusterProfiler 4.0: A universal enrichment tool for interpreting omics data. Innovation *12*, 100141. https://doi.org/10.1016/j.xinn.2021.100141.
52. Xie, Z., Bailey, A., Kuleshov, M.V., Clarke, D.J.B., Evangelista, J.E., Jenkins, S.L., Lachmann, A., Wojciechowicz, M.L., Kropiwnicki, E., Jagodnik, K.M., et al. (2021). Gene Set Knowledge Discovery with Enrichr. Curr. Protoc. *1*, e90. https://doi.org/10.1002/cpz1.90.
53. Heinz, S., Benner, C., Spann, N., Bertolino, E., Lin, Y.C., Laslo, P., Cheng, J.X., Murre, C., Singh, H., and Glass, C.K. (2010). Simple Combinations of Lineage-Determining Transcription Factors Prime cis-Regulatory Elements Required for Macrophage and B Cell Identities. Mol. Cell. *38*, 576–589. https://doi.org/10.1016/j.molcel.2010.05.004.
54. Laurentino, S., Cremers, J.F., Horsthemke, B., Tüttelmann, F., Czeloth, K., Zitzmann, M., Pohl, E., Rahmann, S., Schröder, C., Berres, S., et al. (2020). A germ cell-specific ageing pattern in otherwise healthy men. Aging Cell *19*, e13242. https://doi.org/10.1111/acel.13242.
55. Leitão, E., Di Persio, S., Laurentino, S., Wöste, M., Dugas, M., Kliesch, S., Neuhaus, N., and Horsthemke, B. (2020). The sperm epigenome does not display recurrent epimutations in patients with severely impaired spermatogenesis. Clin. Epigenet. *12*, 61. https://doi.org/10.1186/s13148-020-00854-0.
56. Greenberg, M.V.C., and Bourc'his, D. (2019). The diverse roles of DNA methylation in mammalian development and disease. Nat. Rev. Mol. Cell Biol. *20*, 590–607. https://doi.org/10.1038/s41580-019-0159-6.
57. Piovesan, A., Pelleri, M.C., Antonaros, F., Strippoli, P., Caracausi, M., and Vitale, L. (2019). On the length, weight and GC content of the human genome. BMC Res. Notes *12*, 106. https://doi.org/10.1186/s13104-019-4137-z.
58. Kubo, N., Toh, H., Shirane, K., Shirakawa, T., Kobayashi, H., Sato, T., Sone, H., Sato, Y., Tomizawa, S.i., Tsurusaki, Y., et al. (2015). DNA methylation and gene expression dynamics during spermatogonial stem cell differentiation in the early postnatal mouse testis. BMC Genom. *16*, 624. https://doi.org/10.1186/s12864-015-1833-5.
59. Stadler, M.B., Murr, R., Burger, L., Ivanek, R., Lienert, F., Schöler, A., van Nimwegen, E., Wirbelauer, C., Oakeley, E.J., Gaidatzis, D., et al. (2011). DNA-binding factors shape the

mouse methylome at distal regulatory regions. Nature *480*, 490–495. https://doi.org/10.1038/nature10716.

60. Ball, M.P., Li, J.B., Gao, Y., Lee, J.-H., LeProust, E.M., Park, I.-H., Xie, B., Daley, G.Q., and Church, G.M. (2009). Targeted and genome-scale strategies reveal gene-body methylation signatures in human cells. Nat. Biotechnol. *27*, 361–368. https://doi.org/10.1038/nbt.1533.
61. Hammoud, S.S., Nix, D.A., Zhang, H., Purwar, J., Carrell, D.T., and Cairns, B.R. (2009). Distinctive chromatin in human sperm packages genes for embryo development. Nature *460*, 473–478. https://doi.org/10.1038/nature08162.
62. Brykczynska, U., Hisano, M., Erkek, S., Ramos, L., Oakeley, E.J., Roloff, T.C., Beisel, C., Schübeler, D., Stadler, M.B., and Peters, A.H.F.M. (2010). Repressive and active histone methylation mark distinct promoters in human and mouse spermatozoa. Nat. Struct. Mol. Biol. *17*, 679–687. https://doi.org/10.1038/nsmb.1821.
63. Sharda, A., and Humphrey, T.C. (2022). The role of histone H3K36me3 writers, readers and erasers in maintaining genome stability. DNA Repair *119*, 103407. https://doi.org/10.1016/j.dnarep.2022.103407.
64. Marques, C.J., Carvalho, F., Sousa, M., and Barros, A. (2004). Genomic imprinting in disruptive spermatogenesis. Lancet *363*, 1700–1702. https://doi.org/10.1016/S0140-6736(04)16256-9.
65. Poplinski, A., Tüttelmann, F., Kanber, D., Horsthemke, B., and Gromoll, J. (2010). Idiopathic male infertility is strongly associated with aberrant methylation of *MEST* and *IGF2/H19 ICR1*. Int. J. Androl. *33*, 642–649. https://doi.org/10.1111/j.1365-2605.2009.01000.x.
66. Kläver, R., Tüttelmann, F., Bleiziffer, A., Haaf, T., Kliesch, S., and Gromoll, J. (2013). DNA methylation in spermatozoa as a prospective marker in andrology. Andrology *1*, 731–740. https://doi.org/10.1111/j.2047-2927.2013.00118.x.
67. Kuhtz, J., Schneider, E., El Hajj, N., Zimmermann, L., Fust, O., Linek, B., Seufert, R., Hahn, T., Schorsch, M., and Haaf, T. (2014). Epigenetic heterogeneity of developmentally important genes in human sperm: Implications for assisted reproduction outcome. Epigenetics *9*, 1648–1658. https://doi.org/10.4161/15592294.2014.988063.
68. Laurentino, S., Beygo, J., Nordhoff, V., Kliesch, S., Wistuba, J., Borgmann, J., Buiting, K., Horsthemke, B., and Gromoll, J. (2015). Epigenetic germline mosaicism in infertile men. Hum. Mol. Genet. *24*, 1295–1304. https://doi.org/10.1093/hmg/ddu540.
69. Wang, P.J., McCarrey, J.R., Yang, F., and Page, D.C. (2001). An abundance of X-linked genes expressed in spermatogonia. Nat. Genet. *27*, 422–426. https://doi.org/10.1038/86927.
70. Wang, L., and Wolgemuth, D.J. (2016). BET Protein BRDT Complexes With HDAC1, PRMT5, and TRIM28 and Functions in Transcriptional Repression During Spermatogenesis. J. Cell. Biochem. *117*, 1429–1438. https://doi.org/10.1002/jcb.25433.
71. Ma, P., de Waal, E., Weaver, J.R., Bartolomei, M.S., and Schultz, R.M. (2015). A DNMT3A2-HDAC2 Complex Is Essential for Genomic Imprinting and Genome Integrity in Mouse Oocytes. Cell Rep. *13*, 1552–1560. https://doi.org/10.1016/j.celrep.2015.10.031.
72. Lambrot, R., Chan, D., Shao, X., Aarabi, M., Kwan, T., Bourque, G., Moskovtsev, S., Librach, C., Trasler, J., Dumeaux, V., and Kimmins, S. (2021). Whole-genome sequencing of H3K4me3 and DNA methylation in human sperm reveals regions of overlap linked to fertility and development. Cell Rep. *36*, 109418. https://doi.org/10.1016/j.celrep.2021.109418.
73. Sasaki, T., Nishihara, H., Hirakawa, M., Fujimura, K., Tanaka, M., Kokubo, N., Kimura-Yoshida, C., Matsuo, I., Sumiyama, K., Saitou, N., et al. (2008). Possible involvement of SINEs in mammalian-specific brain formation*105*, pp. 4220–4225. https://doi.org/10.1073/pnas.0709398105.
74. Åsenius, F., Danson, A.F., and Marzi, S.J. (2020). DNA methylation in human sperm: a systematic review. Hum. Reprod. Update *26*, 841–873. https://doi.org/10.1093/humupd/dmaa025.
75. Soumillon, M., Necsulea, A., Weier, M., Brawand, D., Zhang, X., Gu, H., Barthès, P., Kokkinaki, M., Nef, S., Gnirke, A., et al. (2013). Cellular Source and Mechanisms of High Transcriptome Complexity in the Mammalian Testis. Cell Rep. *3*, 2179–2190. https://doi.org/10.1016/j.celrep.2013.05.031.
76. Sangrithi, M.N., Royo, H., Mahadevaiah, S.K., Ojarikre, O., Bhaw, L., Sesay, A., Peters, A.H.F.M., Stadler, M., and Turner, J.M.A. (2017). Non-Canonical and Sexually Dimorphic X Dosage Compensation States in the Mouse and Human Germline. Dev. Cell *40*, 289–301.e3. https://doi.org/10.1016/j.devcel.2016.12.023.
77. Ernst, C., Eling, N., Martinez-Jimenez, C.P., Marioni, J.C., and Odom, D.T. (2019). Staged developmental mapping and X chromosome transcriptional dynamics during mouse spermatogenesis. Nat. Commun. *10*, 1251. https://doi.org/10.1038/s41467-019-09182-1.
78. Raiz, J., Damert, A., Chira, S., Held, U., Klawitter, S., Hamdorf, M., Löwer, J., Strätling, W.H., Löwer, R., and Schumann, G.G. (2012). The non-autonomous retrotransposon SVA is trans -mobilized by the human LINE-1 protein machinery. Nucleic Acids Res. *40*, 1666–1683. https://doi.org/10.1093/nar/gkr863.
79. De Fazio, S., Bartonicek, N., Di Giacomo, M., Abreu-Goodger, C., Sankar, A., Funaya, C., Antony, C., Moreira, P.N., Enright, A.J., and O'Carroll, D. (2011). The endonuclease activity of Mili fuels piRNA amplification that silences LINE1 elements. Nature *480*, 259–263. https://doi.org/10.1038/nature10547.

The American Journal of Human Genetics, Volume *111*

Supplemental information

Genome-wide DNA methylation changes

in human spermatogenesis

Lara M. Siebert-Kuss, Verena Dietrich, Sara Di Persio, Jahnavi Bhaskaran, Martin Stehling, Jann-Frederik Cremers, Sarah Sandmann, Julian Varghese, Sabine Kliesch, Stefan Schlatt, Juan M. Vaquerizas, Nina Neuhaus, and Sandra Laurentino

Supplemental information

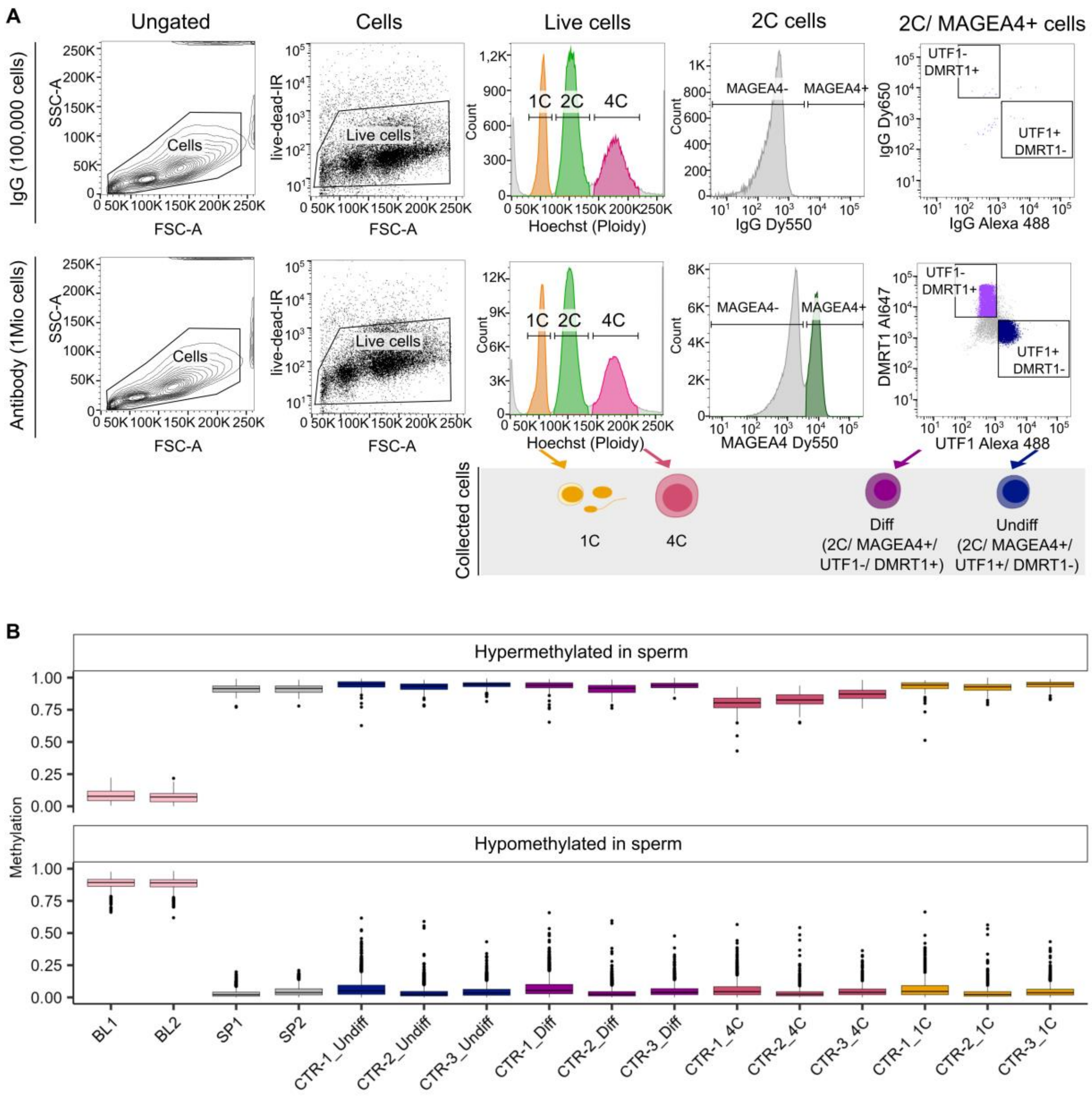

Figure S1: Sorting strategy and quality check of sorted cells. A Sorting strategy for the different germ cell types represented by 100,000 cells in the IgG and 1 million (Mio) cells in the antibodystained cells. IgG control showed no positive signal for MAGEA4 within the 2C cells and no signal for UTF1 and DMRT1 within the 2C/MAGEA4+ cells. **B** Box plots display methylation of sperm and blood[60] and each control (CTR) sample in 2,761 sperm-soma DMRs of which 121 are hypermethylated and 2,640 are hypomethylated in sperm. Data are represented as median (center line), upper/ lower quartiles (box

limits), 1.5 x interquartile range (whiskers). Undiff = undifferentiated spermatogonia, Diff = differentiating spermatogonia, 4C = primary spermatocytes, 1C = spermatids/sperm.

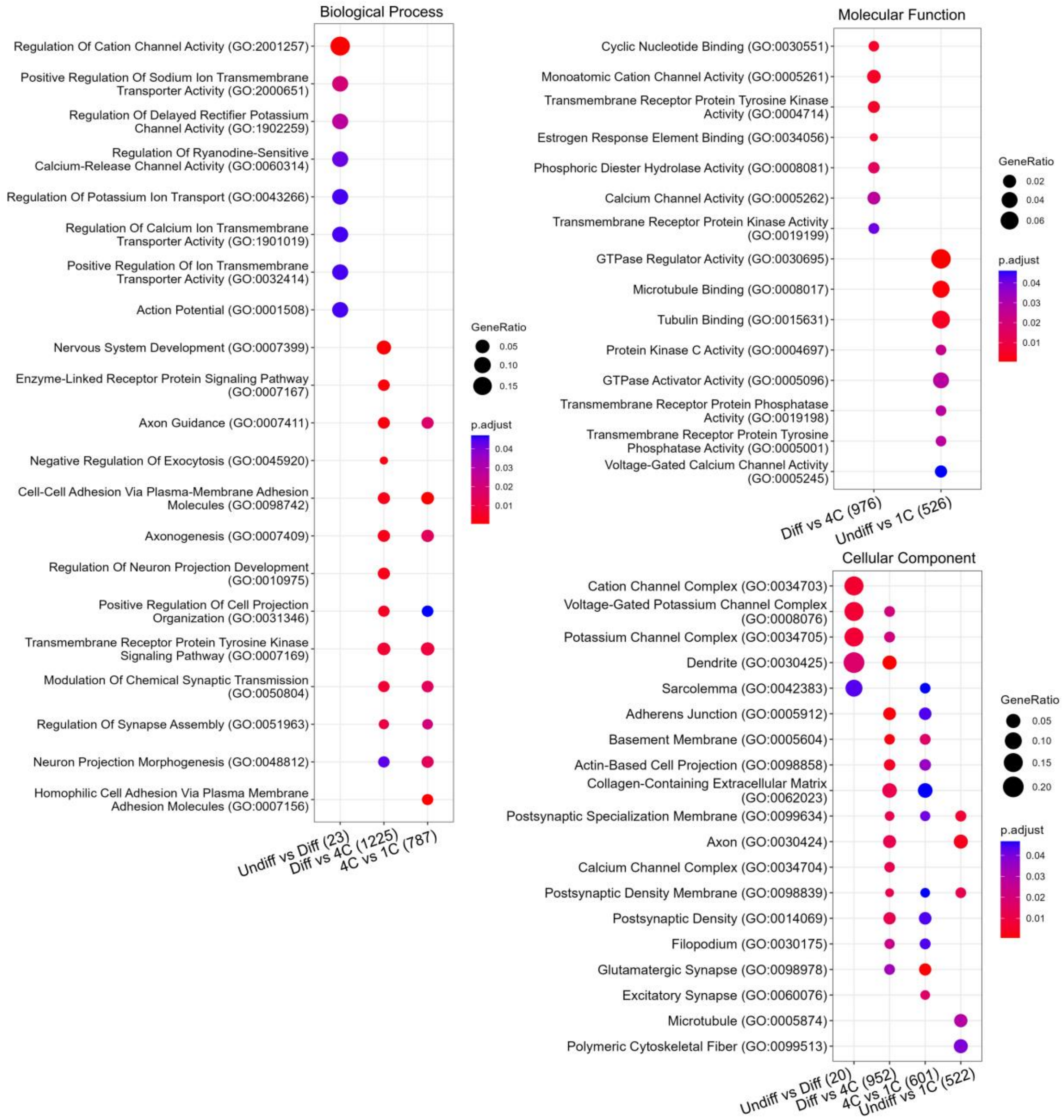

Figure S2: GO term enrichment analysis of biological processes, molecular function and cellular components of the control (CTR) differentially methylated regions (DMRs). All DMR group comparisons were assessed and significant results of the top 8 terms with the lowest p-values are displayed. P-values were adjusted for multiple testing with Benjamini-Hochberg correction. Undiff =

undifferentiated spermatogonia, Diff = differentiating spermatogonia, 4C = primary spermatocytes, 1C = spermatids/sperm.

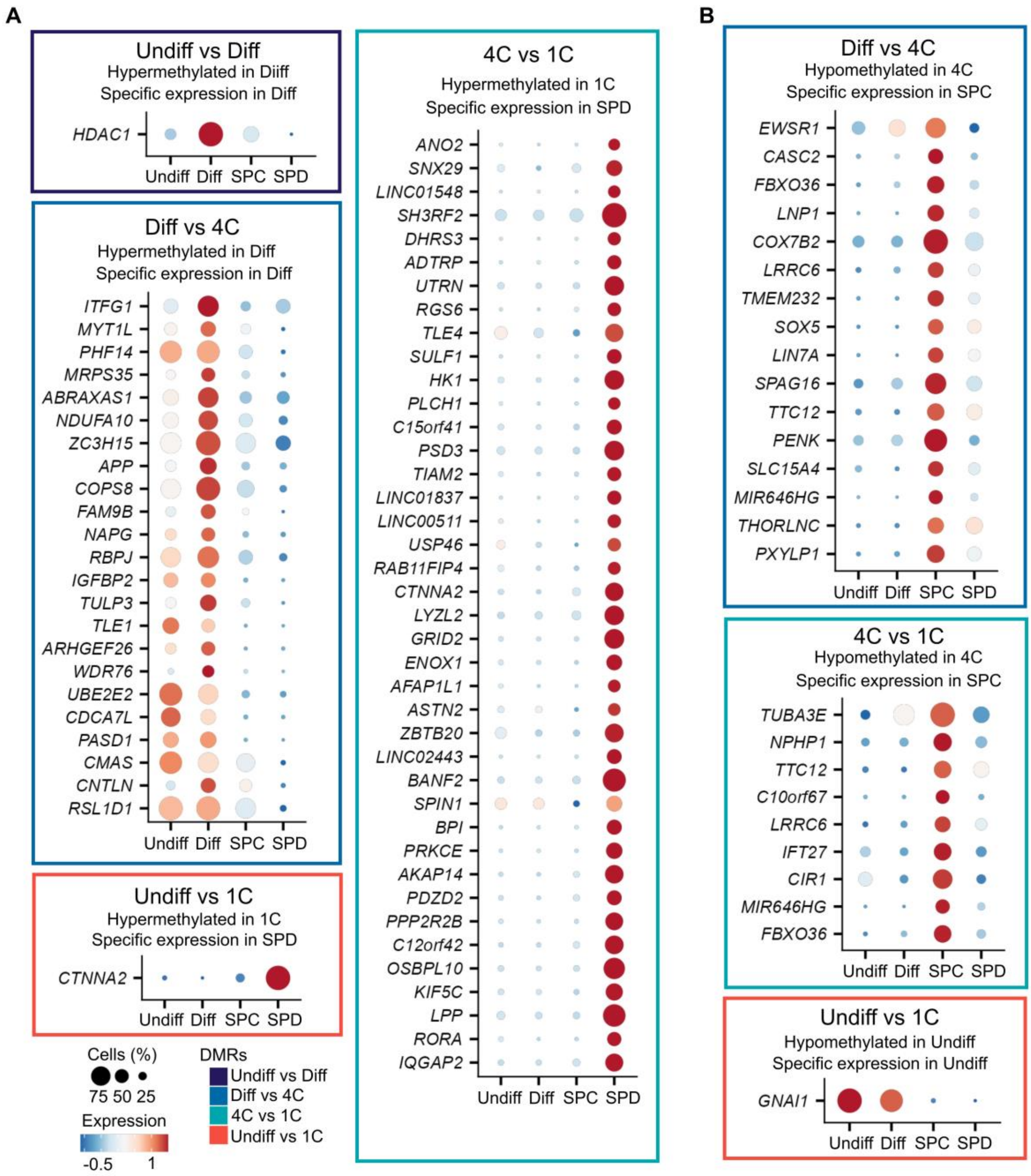

Figure S3: Differentially methylated region (DMR)-associated genes with germ cell type-specific expression. A Hypermethylated and **B** hypomethylated DMR-associated genes of the different group comparisons with specific expression in the respective germ cell types[16] are depicted in dot plots. Undiff = undifferentiated spermatogonia, Diff = differentiating spermatogonia, 4C = primary spermatocytes, 1C = spermatids/sperm, SPC = spermatocytes, SPD = spermatids.

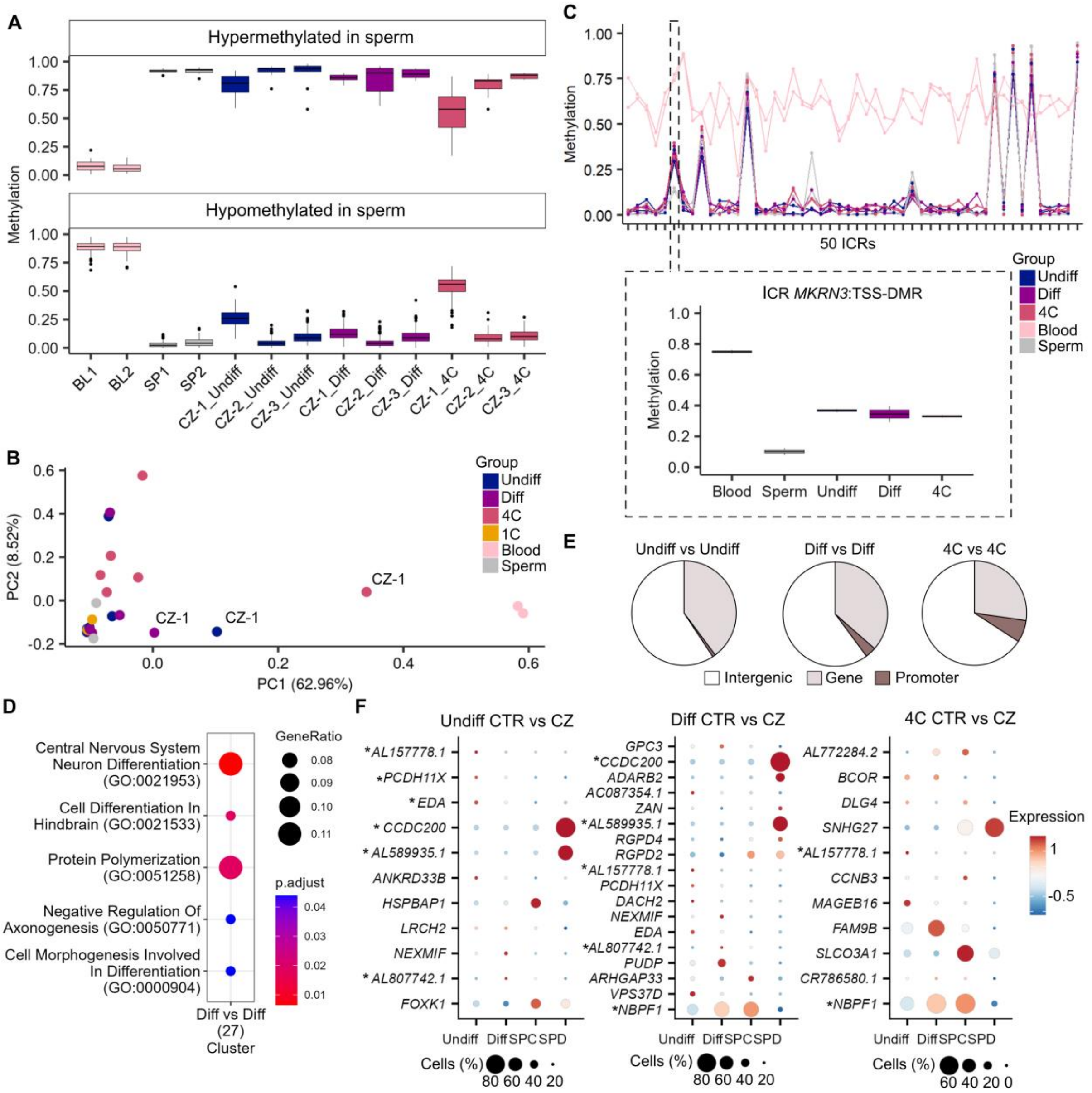

Figure S4: Purity check of the cryptozoospermic samples and features of control (CTR)/cryptozoospermic (CZ) differentially methylated region (DMR)-associated genes. A Box plots display methylation of sperm and blood[60] and each CZ sample in 2,761 sperm-soma DMRs. **B** Principal component analysis (PCA) of 2,521 CpGs of the imprinting control regions (ICRs) depicts clustering of all CTR samples and CZ-2 and CZ-3 samples together with sperm, whereas CZ-1 clustered towards blood, explaining 62.96% of variance (PC1). **C** Lineplots show the mean methylation in the 50 ICRs (Table S8) for CZ-2 and CZ-3 in undifferentiated spermatogonia, differentiating spermatogonia, and primary spermatocytes compared to blood and sperm samples[60]. Box plots display the methylation of

MKRN3:TSS-DMR in the respective cell types. Data are represented as median (center line), upper/ lower quartiles (box limits), 1.5 x interquartile range (whiskers). **D** Piecharts show the annotation of the CTR/CZ DMRs for genes, promoters and intergenic regions. **E** GO term enrichment analysis for biological processes in the CTR/CZ DMRs. The top 5 results are shown. **F** Dot plots showing DMR-associated genes with germ cell-type specific expression[16]. Genes marked with asterisks are present in more than one group. Undiff = undifferentiated spermatogonia, Diff = differentiating spermatogonia, 4C = primary spermatocytes, SPC = spermatocytes, SPD = spermatids. Panel A assembled with BioRender.com.

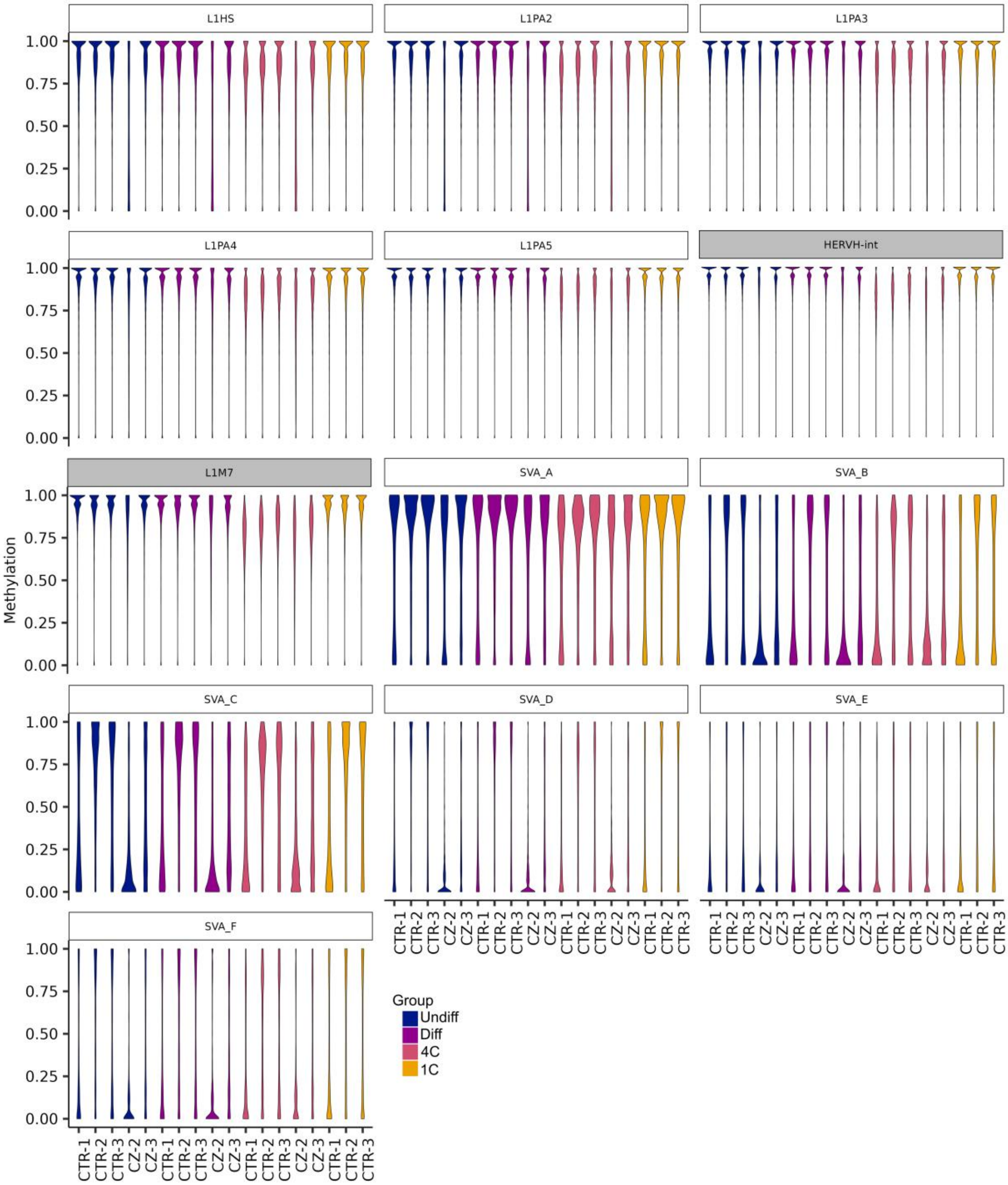

Figure S5: DNA methylation levels of transposable elements (TEs) per control (CTR) and cryptozoospermic (CZ) samples. Violin plots showing the CpG methylation of evolutionarily younger (white boxes: L1Hs, L1PA2-5, and SVA A/B/C/D/E/F) and older (grey boxes: HERVH-int and L1M7) TEs

per CTR and CZ samples. Undiff = undifferentiated spermatogonia, Diff = differentiating spermatogonia, 4C = primary spermatocytes, 1C = spermatids/sperm.

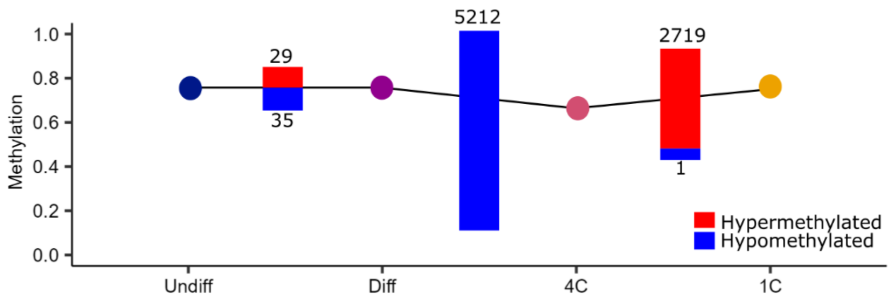

Figure S6: Schematic summary of DNA methylation changes throughout normal spermatogenesis identified in this study. Mean global methylation levels are indicated by dots. Number of hyper- or hypomethylated differentially methylated regions (DMRs), compared to the more advanced germ cell type, are depicted as bars and color-coded red and blue, respectively. Undiff = undifferentiated spermatogonia, Diff = differentiating spermatogonia, 4C = primary spermatocytes, 1C = spermatids/sperm.
